# Human Peripheral Myelin Protein 2 and Charcot–Marie–Tooth Disease or Structural Missense Variants Show Different Binding to Myelin‐Like Lipid Monolayers

**DOI:** 10.1002/cbic.202500947

**Published:** 2026-04-21

**Authors:** Florian Arndt Schöffmann, Md Abdus Shukur Imran, Christian Schwieger, Øystein Hetland, Vanessa Jerschabek, Arne Raasakka, Petri Kursula, Dariush Hinderberger

**Affiliations:** ^1^ Institute of Chemistry Physical Chemistry – Complex Self-Organizing Systems Martin Luther University Halle-Wittenberg Halle (Saale) Germany; ^2^ Faculty of Biochemistry and Molecular Medicine University of Oulu Oulu Finland; ^3^ Department of Biomedicine University of Bergen Bergen Norway; ^4^ LINXS Institute of Advanced Neutron and X-Ray Science Lund Sweden

**Keywords:** epifluorescence, FABP, lipid monolayers, maximal insertion pressure, myelin

## Abstract

The peripheral myelin protein P2 (also known as PMP2/FABP8) has two basic functions in vivo: the transport of fatty acids within the cell to the target compartments and the adhesion of lipid bilayers in the peripheral nervous system myelin to each other. In this work, we focus on the membrane‐binding properties of P2 and its Charcot–Marie–Tooth disease variants. Experiments were carried out on lipid monolayers at the air–water interface as a membrane model system, with the lipid composition of the cytoplasmic leaflet of myelin. Our study provides quantitative data on the membrane affinity of P2 and its disease‐linked or structure‐based missense variants toward the native lipid membrane and shows affinity differences due to single P2 point mutations. Phospholipid monolayer surface pressure measurements are supported by epifluorescence microscopy, which not only shows the adhesive property of all P2 variants but also the complex cross‐linking property of the wild‐type P2. An analysis of transcriptomics databases confirms expression of P2 in human, but not mouse, central nervous system nonneuronal cells. Taken together, our work further confirms the role of P2 in binding myelin‐like lipid membranes as well as its direct effects on lipid membrane properties.

## Graphical abstract

We compare the membrane affinity of the P2 myelin protein and its Charcot–Marie–Tooth disease variants on a myelin‐like lipid monolayer. Epifluorescence microscopy reveals different two‐dimensional (2D) clustering and pressure‐dependent binding. Single‐point mutations in the P2 β‐barrel and α‐helix affect affinity. Transcriptomics confirm high P2 expression in human brain myelinating cells, but not in mouse cells.

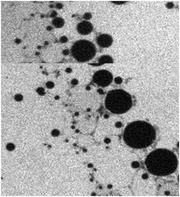

## 1 Introduction

Myelin is a lipid‐rich, insulating layer around the axons of the central nervous system (CNS) and peripheral nervous system (PNS). It enables the rapid saltatory conduction of nerve impulses along the axons. Myelin contains around 75% lipids, with a high cholesterol content of 30%–40%. Schwann cells and oligodendrocytes form and maintain the myelin sheath in the PNS and CNS, respectively. The myelin proteome and its interactions have been the focus of research for decades, but some of its functions and structural connections are still poorly understood. The peripheral myelin protein P2 (also known as PMP2 or FABP8) is part of the myelin proteome [1, 2, 3, 4, 5, 6] and has two basic functions in vivo: (i) the transport of fatty acids within the cell to the target compartments—P2 is a member of the family of fatty acid binding proteins (FABP) as FABP8, and (ii) the adhesion of myelin membranes in the PNS to form multilayers. Thus, it supports the formation of the myelin sheath around axons and promotes saltatory conduction of nerve impulses. Single‐point mutation variants of P2 change myelin such that the native function and the saltatory conduction are hampered, e.g., in Charcot–Marie–Tooth (CMT) disease [7, 8, 9, 10, 11, 12]. The physical effects of P2‐associated neuronal diseases can be described in terms of differences in (i) membrane affinity between the P2 variants, (ii) ligand affinity, and (iii) temperature dependence [7, 8].

P2 binds to lipid membranes and stabilizes them, which was earlier investigated mainly using DMPC/DMPG vesicles. Also, the effect of the lipid composition in the membrane on P2 binding was investigated before [10, 11, 12, 13, 14]. In myelin proteome research, myelin basic protein (MBP) has been studied more intensely than P2. The intrinsically disordered MBP is a major component of the CNS and PNS myelin of humans and other vertebrate species and has a high, charge‐dependent affinity toward myelin‐like mono‐ and bilayer membranes [15, 16, 17, 18, 19, 20, 21, 22, 23, 24].

So far, studies on P2 have mainly used theoretical approaches or greatly simplified model membranes to conclude on membrane affinity. Experimental insights in the context of a more complex, “near‐native” lipid mix—as described here—are needed as an important step toward understanding native function. It is known [5] that P2 binds to the endoplasmic reticulum (ER) membrane of myelinating Schwann cells as well as to the cytoplasmic leaflet of the plasma membrane. This delineates the functioning of P2 as a FABP: transporting fatty acids from the ER to the plasma membrane. P2 binding to the latter will be issue of the present study.

For binding to a native phospholipid bilayer, it is expected that a peripheral membrane protein presents regions with positively charged and hydrophobic residues on its surface. These patches may well be identical to or contain parts of the patches through which FABPs 3, 4, and 5 can attach an intermediately immobilized fatty acid [25]. P2 has two positively charged patches on opposite sides that could be involved in membrane stacking [12]. The helical lid, i.e., the α‐helix structure that seals the ligand‐binding cavity, also contains outward‐facing hydrophobic residues, as possible membrane insertion sites. Prediction of the mode of binding to a single membrane surface [26] suggests partial penetration of the P2 helical lid, which has a hydrophobic tip pointing outward comprising Leu27, Leu32, and Leu35 and a positively charged rim. Membrane binding could lead to lipid transfer to/from the cavity, similar to collisional transfer in other FABPs [27].

P2 binds two membranes simultaneously and can thus build complex membrane structures [12], it was shown that by designing missense variants, the binding behavior can be influenced [28], and there are synergies between three different proteins in membranes considering the binding to membranes [29]. For P2, in addition to the membrane‐binding site on the helical lid [8, 12], a second binding site is located on the opposite side in the loop β5–β6, where Arg88 and Arg78/Lys79/Arg96 form the second anchor for membrane binding [12,30]. This second binding site can interact with negatively charged lipids such as PIP2 (phosphatidylinositol‐4,5‐bisphosphate) and PS (phosphatidylserine), and binding is coupled to an opening of the discontinuous β‐barrel.

Several studies during recent years have focused on P2, which colocalizes with MBP at the major dense line. A range of techniques have been used in this respect, ranging from the molecular to the tissue level. The fact that mutations in the *PMP2* gene are causative of human neuropathy [8, 31,32, 33, 34, 35, 36] and the observations that lack of P2 in mice alters lipid metabolism and remyelination [7, 37] have pointed toward roles of P2 in myelin formation and maintenance. Further studies showed direct binding of lipid membranes by P2 as well as the alteration of membrane structure upon P2 binding. The methods used in the latter studies included, e.g., surface plasmon resonance (SPR), X‐ray and neutron diffraction, coarse‐grained simulations, atomic force microscopy, cryo‐EM, and time‐lapse epifluorescence imaging [11, 12, 14, 20, 31]. In addition, to gain atomic resolution information on P2 structure and dynamics as well as its conformational changes upon membrane binding, a combination of biophysical techniques has been employed, including small‐angle X‐ray scattering, X‐ray and neutron crystallography, neutron scattering, MD simulations, and synchrotron CD spectroscopy [8, 12, 14, 31, 38, 39, 40, 41, 42, 43].

Another method that has successfully been used to study MBP and P0 [22, 44] is neutron reflectometry, which could provide information on the interactions at the membrane surface as well as insertion of P2 into the membrane. In addition, deuterated P2 [40] could be further used in neutron scattering and diffraction studies to observe its association with membrane structure.

In comparison to MBP, P2 associates and dissociates more rapidly with membranes. The formed membrane multilayer is tightly packed, with a repeat distance of ~9 nm [8, 12, 13, 30, 31, 41, 45]. Based on studies on the mouse nervous system, P2 is considered to be specific to selected myelinating Schwann cells in the PNS. However, a recent proteomics study indicated that P2 mRNA and protein are present in the human, but not mouse, CNS myelin [46]. In addition, high expression of P2 has been reported in human, but not mouse, CNS astrocytes [47, 48, 49, 50]. These studies point at possible key differences in the CNS myelin biochemistry between humans and rodents.

We investigated here the binding affinities of P2 and three missense variants toward complex lipid membranes from three perspectives: (i) the maximum insertion pressure (MIP) into lipid monolayers, which provides information about the membrane affinity of the proteins, (ii) the synergy, which provides information about the attraction or repulsion of the lipids and the protein, and finally (iii) epifluorescence microscopy, which delivers insights into larger structures formed through a series of images of the lipid monolayer with and without the proteins in relation to surface pressure. Furthermore, using open transcriptomics datasets, we confirm that P2 is abundantly expressed in the human, but not mouse, CNS.

## 2 Material and Methods

### 2.1 Materials

Human P2 was produced recombinantly in *E. coli* as described [41], dissolved in HEPES/NaCl (10 mM/150 mM) pH 7.4 at 500 µM, and stored at 4°C. The lipids porcine brain L‐α‐phosphatidylcholine (PC), porcine brain L‐α‐phosphatidylserine (PS), porcine brain L‐α‐phosphatidylethanolamine (PE), porcine brain sphingomyelin (SM), bovine liver L‐α‐phosphatidylinositol (PI), and ovine wool cholesterol (ch) were purchased from Avanti Polar Lipids (Alabaster, USA). The fluorescent dye 1,2‐dihexadecanoyl‐sn‐glycero‐3‐phosphoethanol‐amine‐N‐(lissamine rhodamine B sulfonyl) (RhoB−DHPE) (Ex/Em 543/565 nm) was obtained from Life Technologies GmbH (Darmstadt, Germany), and the Alexa‐488‐NHS‐ester (Ex/Em 493/520 nm) was obtained from Fisher Scientific, Leicestershire, UK. HPLC‐grade chloroform was purchased from Carl Roth GmbH & Co. KG (Karlsruhe, Germany). All substances mentioned above were used without further purification.

#### 2.1.1 Lipid Sample Preparation

The lipid mixture in the monolayers has a composition similar to that of the cytoplasmic leaflet of peripheral nerve myelin (cyt‐monolayer), with a molar ratio of ch:PE:PS:PC:SM:PI; 37:22:19:9:9:4 [9, 15]. The lipids were dissolved in chloroform (HPLC grade) and stored at −20°C. The final total lipid concentration of the spreading solutions was 0.5 mM.

#### 2.1.2 Protein Labeling

20 µM wild‐type P2 was incubated for 30 min with 60 µM Alexa‐488‐NHS‐ester dissolved in HEPES/NaCl (10 mM/NaCl 150 mM) with a pH of 8.5, in a volume of 200 µL in a microwell plate. After incubation, the column (packed Sephadex G10 mini column) was equilibrated with 3 × 3 ml HEPES/NaCl buffer (10 mM/150 mM) pH 7.5; then, 200 µL incubation solution and a further 300 µL elution buffer (HEPES/NaCl buffer (10 mM/150 mM) pH 7.5) were added, followed by a further 600 µL elution buffer. The conjugate‐containing fractions were combined, and fluorescence intensity and protein concentration were determined using a NanoDrop spectrophotometer (Thermo Fisher).

### 2.2 Methods

#### 2.2.1 Langmuir Monolayers on Constant Surface Area Langmuir Film Balances

For measurements of MIP and synergy, the surface pressure was detected on two round Teflon‐coated Langmuir troughs. The lipid monolayer was spread dropwise on the buffer surface with a Hamilton syringe. After spreading, chloroform was allowed to evaporate for 20–30 min. The troughs were equipped with a Wilhelmy plate (Riegler & Kirstein GmbH, Potsdam, Germany). Before measurements, the troughs were cleaned with Hellmanex and ultrapure water. All experiments were performed at 20 ± 0.1°C, maintained by a circulating water bath. For protein experiments, a P2 stock solution was injected with a syringe underneath the equilibrated lipid monolayer. Gentle stirring in the solution was achieved through a rotating magnetic stirrer. The final trough concentration of P2 was 200 nM.

#### 2.2.2 Epifluorescence Microscopy on a Compression Langmuir Monolayer Film Balance

For measurements of surface pressure‐area compression isotherms of lipid monolayers, the lipid mixture was spread on a subphase of buffer, with or without protein. A Teflon‐coated Langmuir trough (266 × 99 × 3 mm^3^) was used, and the monolayer was spread dropwise with a Hamilton syringe. After spreading, chloroform evaporation was allowed for 20 min. The trough was equipped with a Wilhelmy plate (Riegler & Kirstein GmbH, Potsdam, Germany) and two symmetrically moveable barriers, which compressed the film in a speed of 2 Å^2^/(molecule·min). Before measurements, the trough was cleaned with Hellmanex and ultrapure water. All experiments were performed at 20 ± 0.1°C. The overall lipid content was held constant at 27.9 nmol to start all experiments in the gas analog phase of the monolayer. For protein experiments, a P2 stock solution was injected with a syringe through the equilibrated lipid monolayer at five different positions to achieve an even distribution in the buffer subphase. The final trough concentration of P2 was 200 nM. P2 was injected at a surface pressure of 20 mN/m, while the monolayer was in the liquid‐expanded (LE) phase.

Images of fluorescent monolayers at the air–water interface were recorded with an Axio Scope A1 Vario epifluorescence microscope (Carl Zeiss MicroImaging, Jena, Germany), while simultaneously recording the compression (surface pressure and area) of the monolayer. The film balance (see above) below the microscope was mounted on an x‐y‐z stage (Märzhäuser, Wetzlar, Germany), which was motion‐controlled by a MAC5000 system (Ludl Electronic Products, Hawthorne, NY, USA). To ensure a dust‐free environment and minimize water evaporation, the trough was enclosed in a custom‐built acrylic glass encasement. The microscope was equipped with a compact light source HXP 120 C (mercury short arc reflector lamp), a long working distance objective (LD EC Epiplan‐NEOFLUAR 50×), and a filter/beam splitter combination appropriate for the fluorescent dyes (all from Carl Zeiss MicroImaging, Jena, Germany). Image data were recorded with an EMCCD camera (ImageEM C9100‐13, Hamamatsu, Herrsching, Germany) and acquired using the AxioVision software (Carl Zeiss MicroImaging, Jena, Germany). All presented images show areas of individually contrast‐adjusted raw data. Each lipid mixture was doped with 0.05 mol% Rh‐DHPE (reducing the amount of brain PE by 0.05 mol%). For experiments with Alexa‐488‐labeled P2 protein, 10 mol% of the overall P2 content was replaced by labeled P2. The excitation wavelengths for the so‐called green channel are 547 nm and for the blue channel 486 nm. The combination of Alexa 488 and rhodamine B DHPE is based on the overlap of the absorption and emission bands of rhodamine DHPE in the Alexa 488 range in the blue channel (Alexa 488: ∼490/513 nm excitation/emission maximum [51]; Rho‐B‐DHPE: ∼560/580 nm excitation/emission maximum [52]). This overlap, which is normally considered negative, enables the localization of labeled proteins within the monolayer in these experiments. This is necessary because lipid monolayers are not rigid systems, and there is continuous movement of the film within the image.

#### 2.2.3 P2 Expression Analysis in Transcriptomics Databases

P2 is encoded by the *PMP2* gene. Brain Knowledge Platform (BKP) in Allen Brain Cell (ABC) Atlas is an integrated platform of massive, multiplexed datasets, which provides a unique opportunity to explore and analyze multimodal single‐cell transcriptomics data across the mammalian brain. This platform is accessible at https://knowledge.brain‐map.org/abcatlas. The Mouse Whole Brain Atlas is a comprehensive high‐resolution dataset of single‐cell RNA sequencing (scRNA‐seq) across the entire mouse brain that uncovers the gene expression and coexpression patterns for each cell type. The 10× scRNAseq NN‐IMN‐GC datasets of mouse whole brain contain a mixed collection of distinct nonneuronal cell types, immature neuronal types, and granule cell types, and cells are grouped by their class [53]. The ABC Atlas also includes datasets of cellular diversity in the human brain, consisting of over three million cells analyzed through single‐nucleus RNA sequencing from the adult human brain, and it includes two tSNE plots—one plot contains approximately 900 000 nonneuronal cells and the other contains approximately 2.5 million neuronal cells [54] . In human neuronal and nonneuronal databases, cells are organized as clusters and superclusters [54]. The human M1 10× dataset includes single‐nucleus transcriptomes from 76,533 total nuclei derived from two postmortem human brain specimens, to survey cell type diversity in the primary motor cortex, while the mouse whole cortex and hippocampus 10× dataset includes single‐cell transcriptomes from multiple cortical areas and the hippocampal formation. The above datasets were used to analyze the expression of *PMP2* in different cell types in human and mouse CNS. In addition, the BrainRNAseq [48, 49] (https://brainrnaseq.org/) database was used to visualize differences in gene expression across human and mouse CNS cell types.

## 3 Results and Discussion

### 3.1 P2 and Missense Variant Interaction with Model Membranes

**FIGURE 1:**
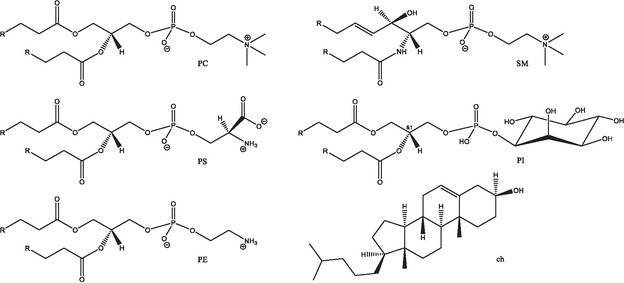
Chemical structures of the typical human PNS membrane lipids: *L‐*α‐phosphatidylcholine (PC), *L*‐α‐phosphatidylserine (PS), *L*‐α‐phosphatidylethanolamine (PE), sphingomyelin (SM), *L*‐α‐phosphatidylinositol (PI), and cholesterol (ch); R stands for a variable lipid chains.

In the current work, we first address the question of how human P2 and its missense variants interact with model membranes with the lipid composition of the cytoplasmic leaflet of the PNS myelin. The lipid mixture described by Inouye and Kirschner ^[9]^ was used to reconstruct the cytoplasmic side of human PNS myelin: ch:PE:PS:PC:SM:PI = 37:22:19:9:9:4 (see Figure 1). The cytoplasmic environment was modeled using the HEPES/NaCl (10 mM/150 mM) buffer [7]. The combination of buffer and lipid mix should reproduce the endogenous environment of the protein.

### 3.2 Maximum Insertion Pressure Measurements

To understand the interaction between P2 or its variants and the PNS myelin cytoplasmic leaflet, monolayer experiments on Langmuir film balances were carried out [55, 56]. Proteins were injected underneath a lipid monolayer of an initial surface pressure, π_ini_, and the resulting surface pressure increase Δπ due to protein insertion is recorded $\text{Δ}\pi =\pi_{\mathrm{end}}-\pi_{\mathrm{ini}.}$

**FIGURE 2:**
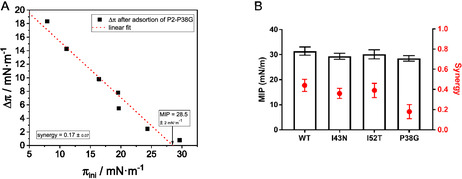
(A) Diagram of Δπ vs. $\pi_{\mathrm{ini}}$, measured on a film balance, here the injection of P2 P38G variant underneath a PNS‐like lipid monolayer as an example. Black points are experimentally determined; the red dotted line is the linear regression line, which intersects the abscissa at the MIP. The synergy is calculated from the slope of the regression. Errors are given as 95% confidence intervals. (B) MIPs and synergies of P2 and its CMT disease variants toward PNS myelin‐mimicking lipid monolayers.

Plotting Δπ as a function of π_ini_ yields the linear relationship Δπ (π_ini_) = *a* + *b* ⋅ π_ini_. The MIP describes monolayer surface pressure, at which the protein can no longer be incorporated into or adsorbed to the lipid monolayer membrane, i.e., Δπ(MIP) = 0 mN/m. The MIP can, thus, be calculated as MIP = − a/b. Hence, the MIP can be determined from a plot of Δπ over π_ini_ as the intersection of the regression line and the *x*‐axis (see, as an example, the data for P2 P38G in Figure 2A; all surface pressure curves and derived Δπ‐diagrams are shown in Figure S1).

Proteins or peptides with a high affinity toward the lipid membrane generally have an MIP of at least 30 mN/m, a value that is usually denoted as the monolayer–bilayer equivalence pressure. For example, bovine myelin basic protein (bMBP) with our myelin‐like lipid monolayer has an MIP of 42 mN/m [18]. Unlike MBP, which in vivo shows posttranslational changes with different affinities and structures in membranes [17, 57], P2 in vivo shows variants that are derived from point mutations. The diagram in Figure 2B summarizes the MIPs and synergies (see next section) of the P2 variants toward a PNS myelin‐mimicking monolayer. While wild‐type P2 has the highest value of the variants with an MIP of 31 mN/m, the membrane affinity of the three missense variants tested in this study is not significantly different from that of the wild‐type protein (MIP = 28.5–31 mN/m). One should note that in SPR studies on immobilized, simplified PC:PG membrane models, the WT P2 in comparison to other disease variants showed fastest and most complete binding to the membranes, which does not seem to be reflected—within the experimental margins of error—in the MIP order found here [8]. The structure‐based variant with the mutation P38G has the lowest MIP (28.5 mN/m), which indicates a lower membrane affinity [13] toward the native‐like myelin membrane model. Point mutant variants show a measurable difference in membrane affinity, which in the case of P38G represents a mutation replacing the rigid proline with a flexible glycine at a structural hinge segment, possibly required for conformational adaptation upon membrane binding. However, it should be noted that the differences in MIP values are small and within experimental error.

### 3.3 Synergy

The so‐called synergy is another indicator of protein–lipid interactions that can be gained from Langmuir film balance adsorption measurements. It is derived from the slope (Δπ(π_ini_)  =  *a* + *b* ⋅ π_ini_) of the regression lines (Figure 2A) and describes the interaction intensity between the lipids of the monolayer and the protein.

synergy=b+1

A positive synergy indicates an attractive interaction, while a negative synergy indicates a repulsive effect between the lipid monolayer and the protein [58].

As expected, the synergy between the lipids and the wild‐type protein has the highest value. The CMT variants I43N and I52T have only slightly lower synergy values, which shows that although the mutation leads to a change in affinity, this is only minute (synergies all around 0.40). The synergy value for P38G is 0.18 and thus clearly smaller than the synergies of the other variants.

The P38G mutation lies between the α‐helical “lid” and the β‐barrel. Proline and glycine both are neither ionic nor very lipophilic amino acids, so a local electrostatic effect or lipophilicity effect can be discarded as explanation. Proline is known to increase structural rigidity clearly when compared to glycine, and thus, the mutation most likely affects P2 functional dynamics, especially in the portal region, as seen before [13, 30, 45]. In a previous study, an increased lipid membrane stacking capacity of P38G was found in vitro, as well as increased dynamics of individual P38G molecules, in particular on the 1 ns scale (through elastic incoherent neutron scattering, EINS) [13]. Since the synergy in case of the P38G variant is lower, the binding to the monolayers is clearly less favored. Whether the better lipid bilayer stacking behavior is related to the increased lid or overall dynamics, which could, e.g., reduce clustering strength laterally, remains unanswered at this point and needs to be addressed in future studies.

### 3.4 Epifluorescence Microscopy

Epifluorescence microscopy was used to illustrate the lateral organization of myelin‐like lipid monolayers with and without adsorbed P2 variants, at a micrometer resolution. The experimental setup contains a fluorescence microscope that is mounted above the Langmuir trough of a compression film balance. Rho‐DHPE was used as a fluorescent dye; this phosphatidylethanolamine has a head group labeled with the fluorescent dye rhodamine B. Rho‐DHPE is preferably incorporated into the LE phase of the monolayer. Thus, these represent the brighter areas in the micrographs [59]. Pretests were carried out to rule out a significant influence of the Rho‐DHPE on the monolayer behavior, including measurements of pressure‐area isotherms (Figure S2). All isotherms for all combinations of variants, labeled variants, with the lipid mixtures are also shown in the SI (Figures S2–S11). In the following, the images of the pure cytoplasmic PNS myelin lipid mix with 0.05% Rho‐DHPE are shown first, followed by measurements in the presence of P2.

Furthermore, we performed experiments that—in addition to Rho‐DHPE—contained P2 and its variants labeled with Alexa‐488, which is excited at a wavelength of 490 nm, clearly separable from RhoB‐DHPE excitation of 560 nm. This makes it possible to study the localization of the adsorbed proteins within the laterally phase‐separated lipid monolayer membrane.

### 3.5 Fluorescence Microscopy of the PNS Lipid Mixture with and without P2 Variants

**FIGURE 3:**
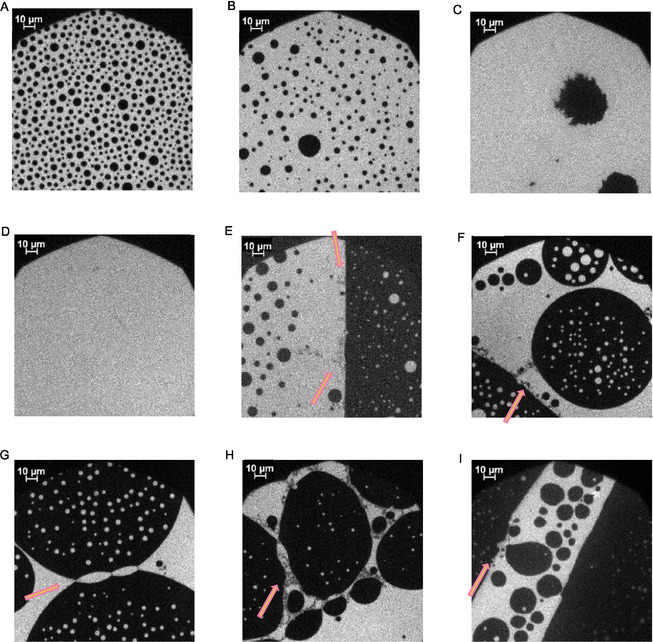
Representative epifluorescence microscopic images of the compression behavior of the PNS myelin‐like monolayer mixed with 0.05% Rho‐DHPE on a HEPES‐NaCl buffer subphase before (A–D) and after (E–I) addition of wild‐type P2 at 20 mN/m and incubation; A, 3.9 mN/m; B, 11.5 mN/m; C, 13.5 mN/m; D, 20 mN/m; E, 22 mN/m; F–I, ∼ 25 mN/m. Arrows indicate regions of reduced Rho‐DHPE fluorescence (“gray veil”) due to clusters including P2. The corresponding pressure‐area isotherm is found in Figure S3.

According to Inouye and Kirschner [9], the cytoplasmic PNS myelin leaflet contains 37 mol% cholesterol. This high proportion has a condensing effect on the lipid mixture [60, 61], influencing the freedom of movement of the phospholipid hydrocarbon chains and thus the overall membrane fluidity. Hence, the phospholipids in such a monolayer occupy less area at the interface, which leads to a reduction in the degree of freedom of lipid movement [62, 63]. Cholesterol also interrupts head group–head group interactions between phospholipids [64]. Overall, it is therefore assumed that these effects facilitate lipid domain formation, leading to domains that are enriched in cholesterol, saturated phospholipids, and sphingolipids, as well as domains depleted in these molecules. The cholesterol‐rich domains are more densely packed, tend to have reduced fluidity [65, 66], can be described as being in the liquid‐ordered (*L*_o_) phase, and can be observed as darker areas in fluorescence micrographs (Figure 3). Monolayer experiments can mimic the native myelin bilayer. This pseudo‐binary system has been intensively investigated [15, 66]. Experiments with TopFluor cholesterol, a fluorescently labeled cholesterol, supported this assumption and showed that the rafts are predominantly enriched with cholesterol. Due to its molecular structure, Rho‐DHPE tends to be depleted in these *L*_o_ phases and enriched in the areas of the liquid‐disordered (*L*_d_) phase. All respective combinations without (Figure S11) and with all P2 variants (Figures S12–S15) are shown in the SI.

The transition between the two‐phase and single‐phase lipid systems is pressure‐dependent. Several domain patterns are possible in the transition states. Circular domains appear at lower surface pressures. Stripes or angular domains follow, which are more typical for higher surface pressures, below the critical mixing pressure [61]. Above a certain pressure, here at ∼20 mN/m, the domain boundaries dissolve, and the heterogeneous monolayer subsequently transforms into a homogeneously mixed monolayer. This leads to the disappearance of the interspersed cholesterol‐rich domains and the formation of a gray homogeneous surface in the micrograph. This homogeneous mixing remains upon further compression and increase in pressure, until the monolayer collapses.

The “patchwork quilt” (Figure S15) of highly ordered, cholesterol‐rich domains and the lower‐order, phospholipid‐rich domains combines many individual images of the surface to show how representative the structures depicted are. Over the entire film, one can observe areas where the domains are enriched and areas where there are hardly any dark domains. This “patchwork” is not surprising, as the distribution of lipids and domains is also inhomogeneous in the natural cell membrane, e.g., described in the fluid–mosaic membrane model (F‐MMM) [67, 68]. More details are given in the beginning of the SI.

**FIGURE 4:**
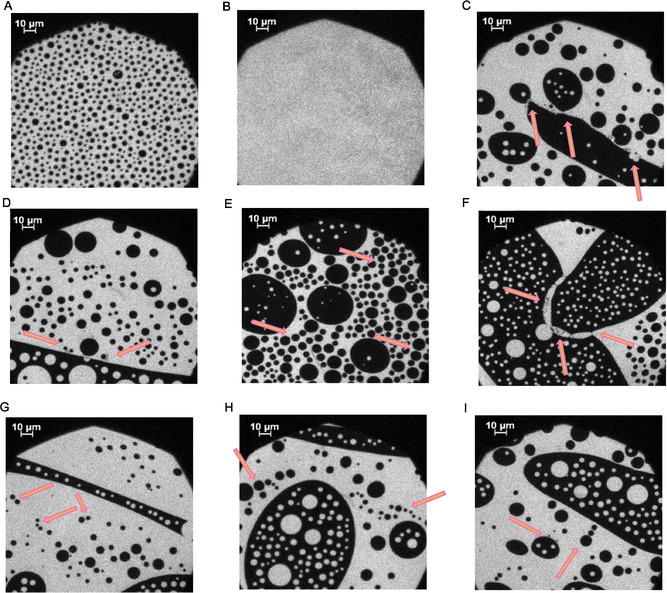
Representative epifluorescence microscopic images of the compression behavior of the PNS myelin‐like monolayer mixed with 0.05% Rho‐DHPE on a HEPES‐NaCl buffer subphase before (A, B) and after (C–I) addition of P2 P38G variant at 20 mN/m and incubation; A, 0.6 mN/m; B, 17.19 mN/m; C–I, ∼25 mN/m. Arrows generally indicate regions of reduced Rho‐DHPE fluorescence (“gray veil”) due to clusters including the P2 variant. In G, H, I, strings of dark areas resembling a string of pearls are also shown. These domains are presumably connected by clusters of P2 P38G. The corresponding pressure‐area and adsorption isotherm is shown in Figure S6.

After incubation with the proteins (Figure 3), an effect can be observed on the monolayer [14]. At the surface pressure of 22 mN/m and higher, light (Rho‐DHPE‐containing) and dark domains have formed. These domains are often not evenly distributed in the plane, but they are clustered or connected with small “veils.” In direct comparison to the micrographs without P2, one may deduce that these “veils” are assemblies of P2 within the monolayer, similar to those described before for bMBP [17] and possibly similar to the P2 lattice observed between two bilayers by cryoEM [30]. P2 can stack DMPC/DMPG bilayers as well as influence the composition of the lipids around its binding site and thus construct a micromilieu [10, 11, 14]. The “gray veils” indicate that the clustered proteins reduce Rho‐DHPE fluorescence emission at these sites. Since these aggregates or clusters are located primarily at the boundaries of cholesterol‐rich and cholesterol‐depleted regions, it can be assumed that P2 reduces the interfacial tension in a way similar to that observed for MBP [18, 19]. In Figure 3G, two dark domains are connected at three anchor points. These anchor points can in the light of the epifluorescence micrographs be identified as clusters of P2. Earlier findings [10, 11] showing P2 binding to vesicles, vesicle aggregation, and multilayer formation by adding P2 to lipid vesicles thus seem to be accompanied by these epifluorescence images of complex lipid mixtures in two dimension. Similar clustered structures can also be observed for the missense variants (I43N: Figure S12, I52T: Figure S13, P38G: Figure 4).

### 3.6 Fluorescence Microscopy of Fluorescently Labeled P2 Variants in the PNS Lipid Mixture

Above, P2 was detected indirectly through diminished fluorescence at the interfaces between lipid domains, a finding reminiscent of the “gray veil” of clustered MBP described by us earlier [18, 19]. The labeling of lipids in a monolayer only allows detection when sufficient amount of dye is displaced, for example, by highly ordered domains or protein clusters. However, a single protein molecule, particularly a small protein like P2 (14.8 kDa) [69], with a maximum diameter of 4–5 nm, is impossible to detect by this method.

**FIGURE 5:**
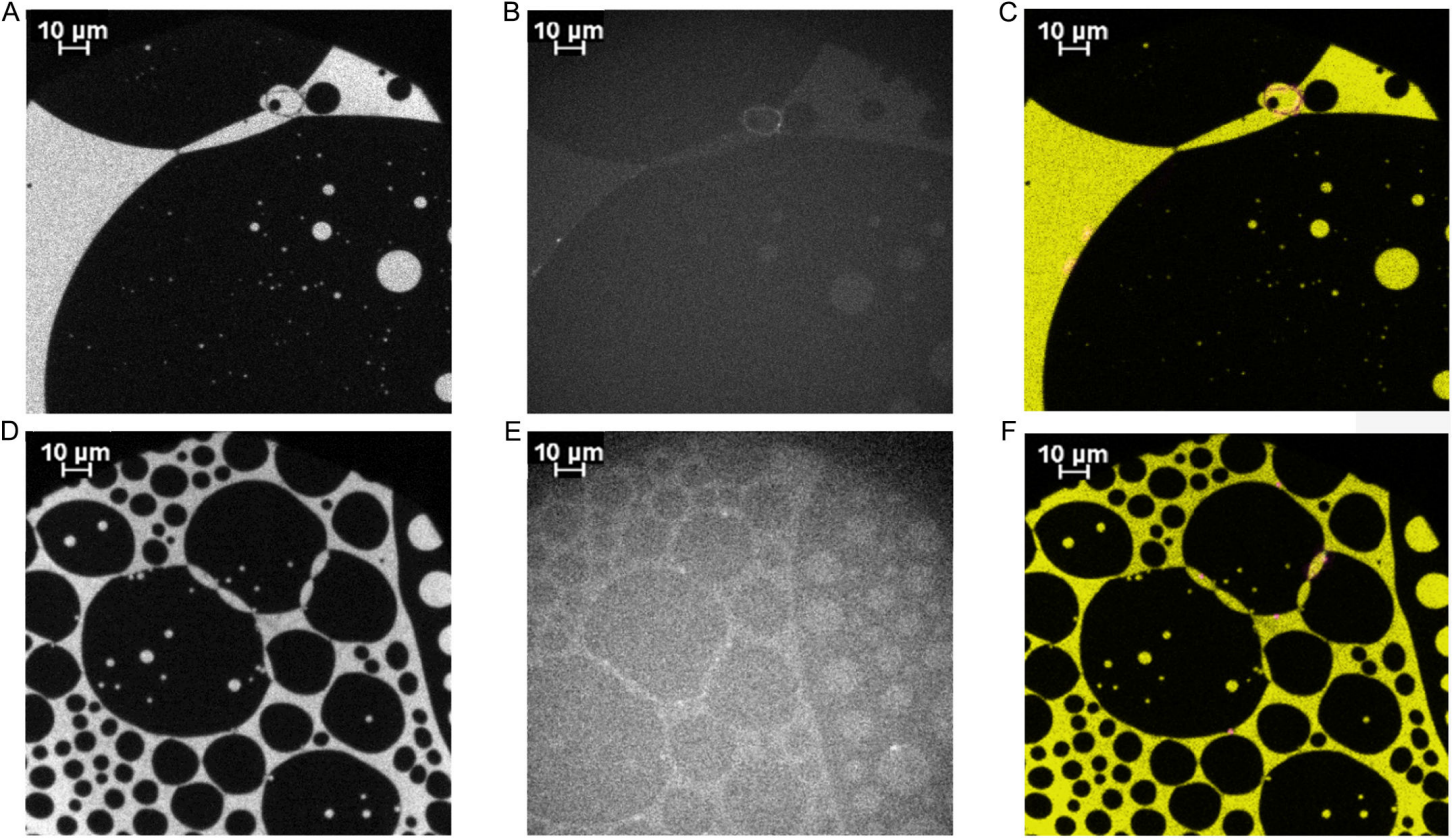
Two‐color experiment of P2 variants in epifluorescence film balance microscopy. (A, B, C) P2 wt; (D, E, F) P2 P38G; A, D, green channel (546 nm excitation wavelength); B, E, blue channel (486 nm excitation wavelength) in myelin‐like monolayers at 25 mN/m; C, F, merged images, false coloring for better differentiation: green channel in yellow and blue channel in magenta. The corresponding compression and adsorption isotherms are shown in Figure S3 (P2 wt) and Figure S6 (P2 P38G).

In order to make small protein aggregates visible and to characterize the interactions between proteins and the respective lipid domains more precisely, P2 was labeled with the fluorescent dye Alexa 488. This dye was chosen because Rho‐DHPE (within the lipid monolayer LE phase) exhibits weak fluorescence when excited together with Alexa 488 with blue light and thus provides a suitable background for the detection of proteins. To minimize the influence of the labeling on the system, only 10% of P2 was replaced by the respective Alexa 488 conjugate. This experimental setup allowed clearly identifying P2 by its fluorescence label instead of only indirectly detecting it by labeled lipid depletion as “gray veil” (Figure 5 and Figure S14).

The fluorescence data corroborate and extend the above results: P2 preferentially binds to the domain boundaries between the phospholipid‐rich and cholesterol‐rich domains. This suggests that the highly ordered, cholesterol‐rich domains are not preferred for P2 binding. Both wild‐type P2 and all variants appear to locate at the boundary line between the *L*_o_ and *L*_d_ phases, which could be relevant for the function of P2 in myelination. This may be related to our earlier observations that P2 induces crystalline structures in simple lipid model systems [30].

Figure 5 (completed in Figure S14) shows examples of measurements with fluorescent Rho‐DHPE and labeled P2 for all variants. The green channel (excitation at 546 nm) shows bright phospholipid‐rich and dark cholesterol‐rich domains, with gray cluster structures representing P2, as described above. Fluorescence detection of the protein (Alexa conjugate, blue channel (excitation at 486 nm)) confirms the localization of P2 at the interface between the domains, which appear to be connected by the protein. In the combined image, the “veils” in the images marked with Rho‐DHPE correspond to the bright areas in images marked with Alexa‐488‐labeled P2, which is a strong indication of the localization of P2 in these areas.

### 3.7 Network/Cluster Formation by Myelin Protein P2

**FIGURE 6:**
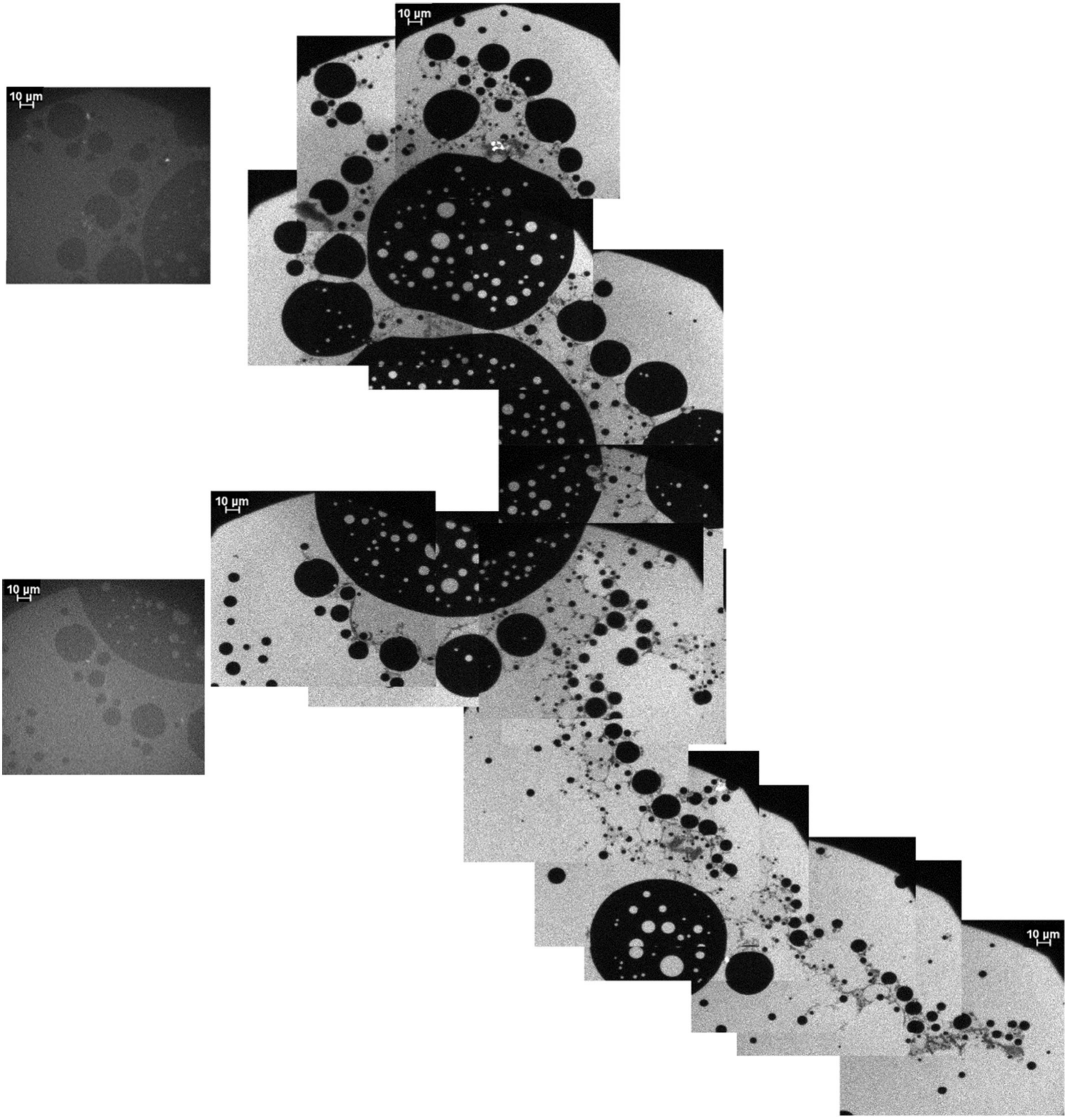
Epifluorescence microscopy of wild‐type P2 (mixed with P2wt‐Alexa) adsorbed to a PNS myelin‐like monolayer marked with Rh‐DHPE. Image series to illustrate the complex cross‐linking of the respective domains by the protein. To the left, two figures with the fluorescent channel of fluorescently labeled P2 are shown, again indicating coincidence of “gray veils” in Rho‐DHPE fluorescence with regions containing labeled P2.

During the series of measurements, wild‐type P2 was the only variant forming large‐scale cluster structures. The variants mainly showed smaller clusters or localized interweaving. The small network structures connected a maximum of three lipid domains with each other, and only wild‐type P2 showed network structures over several domains (Figure 6). This observation was confirmed over multiple measurements. To rule out the possibility that an impurity or surfactant was responsible for this cross‐linking, these experiments were repeated with labeled P2. As shown in Figure 6, labeled P2 can be localized within the observed network. This network structure remained stable over the time of the measurement; reassembly of the domains was not detectable. This may be correlated with the in vivo function of P2 to cross‐link membrane leaflets, which may be one of the stabilizing factors for the myelin sheath. This series of measurements can therefore be taken as further fundamental evidence on the ability of P2 to affect lipid membrane properties and morphology [30, 31].

### 3.8 Analysis of Transcriptomics Databases for P2 Expression in Human Brain

As P2 was long considered to be PNS‐specific, in light of recent data showing its mRNA expression and protein presence in human but not mouse CNS myelin [46], we expanded our study of P2 and its variants with PNS lipids further and analyzed transcriptomics databases to shed light on the expression of the *PMP2* gene in human and mouse brain. This will be taken up further in future studies, as one has to take into account potentially different functions of P2 in human CNS and PNS.

**FIGURE 7:**
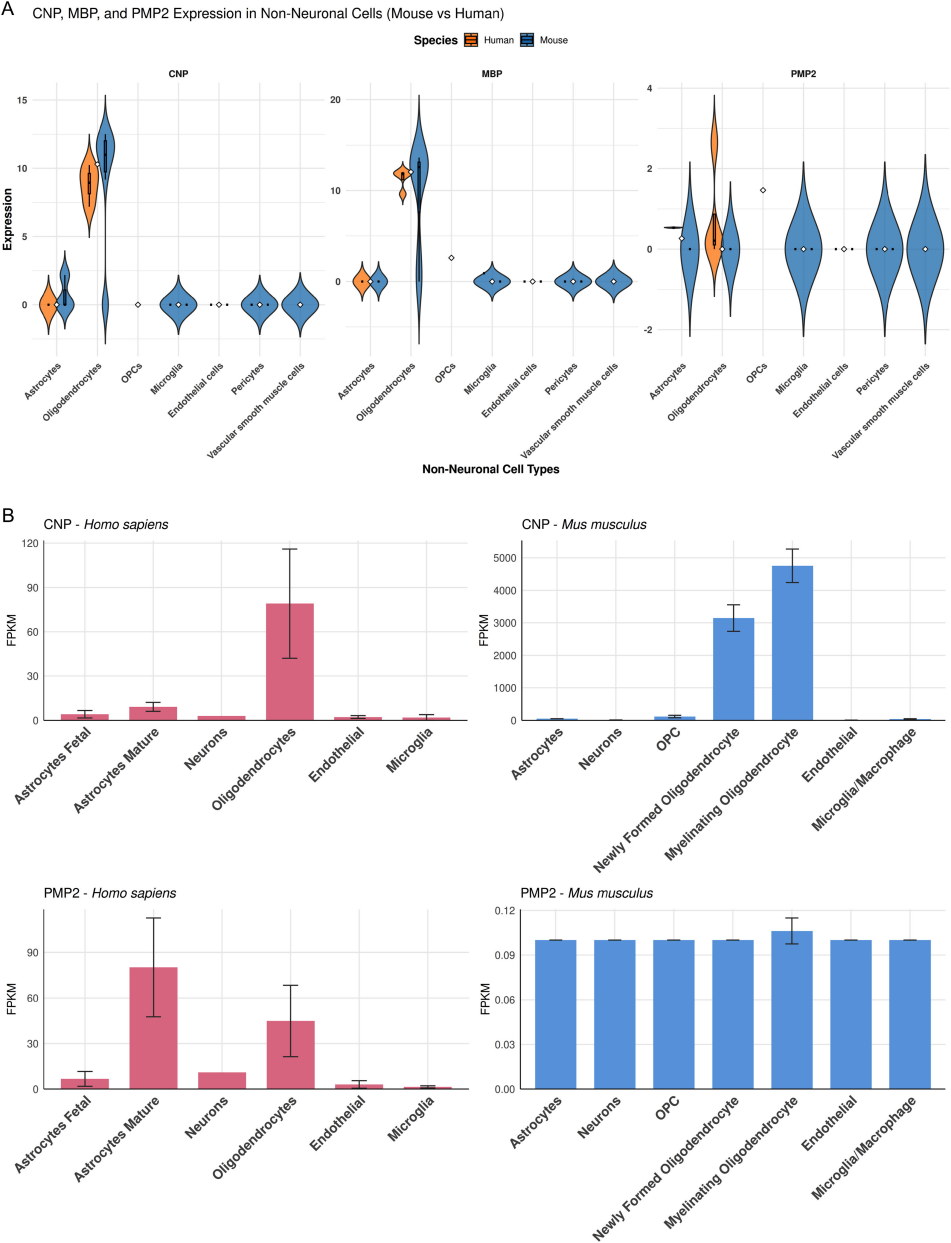
P2 is expressed in human, but not mouse, CNS. (A) *CNP*, *MBP*, and *PMP2* expression in human (orange) and mouse (blue) nonneuronal CNS cell types. A subset of oligodendrocytes in humans expresses *PMP2*. The data for the violin plots are from the human M1 10× and mouse whole cortex and hippocampus 10× datasets. See complementary data in Figure S16. (B) *CNP* and *PMP2* mouse and human gene expression from BrainRNAseq database. Observe *PMP2* expression in human astrocytes and oligodendrocytes, but not in mouse.

The myelin marker genes *MBP* and *CNP* exhibit high levels of expression in nonneuronal cells, especially oligodendrocytes, for both human and mouse CNS (Figure 7A,B). By comparing the gene expression patterns in human nonneuronal cells with the mouse 10× scRNA‐seq NN‐IMN‐GC datasets, we found that the expression of *PMP2* is nearly absent in various nonneuronal cells in mouse CNS, while it is distinctly higher in human CNS (Figure 7A). Data visualization URLs and the single‐cell transcriptomics maps are presented in the SI (Figure S16). Furthermore, using the BrainRNAseq database [48, 49], we found that the expression of *PMP2* in human astrocytes and oligodendrocytes cells is around 80 fragments per kilobase of transcript per million mapped fragments (FPKM) and 41 FPKM, respectively, with the highest levels observed in the nonneuronal cell types. Again, the *PMP2* expression pattern in human nonneuronal cells closely resembles that of the myelinating marker gene *CNP*. Conversely, mouse *PMP2* exhibits distinctly lower level of gene expression (0–0.1 FPKM) compared to the expression of *CNP* (Figure 7B). Thus, the different databases converge on a relatively high expression of *PMP2* in the human CNS, which needs to be taken into account in future research both in vitro and in vivo.

## 4 Conclusion

**FIGURE 8:**
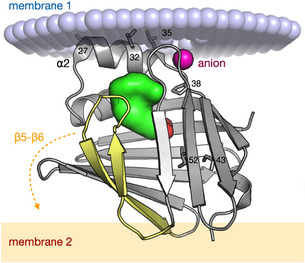
Model for P2 between two membranes. The helical lid and anion‐binding site interact with the membrane at the top (blue spheres), while the β barrel, with a conformational change in the β5‐β6 hairpin (yellow), binds to the bottom membrane (orange box). Locations of the Leu residues 27, 32, and 35 (as sticks) on the outside of helix α2, as well as the mutation sites Pro38, Ile43, and Ile52 are indicated by numbers.

Our study demonstrates that in addition to simple model lipid membranes [12, 13, 30, 41, 45], P2 also adsorbs to lipid monolayers of complex, native‐like composition of the PNS myelin cytoplasmic leaflet. Earlier work has shed light on molecular mechanisms and residues important for P2 interaction with lipid surfaces and stacking into multilayers. Structure‐based mutagenesis coupled with cell‐based assays, cryo‐EM, and atomistic simulations revealed two membrane‐binding surfaces on the P2 protein [12, 28, 30, 31]. As seen in Figure 8, one of the membranes is bound by the helical lid domain of P2, with Leu27, Leu32, and Leu35 possibly inserting into the membrane. Close to this surface, X‐ray crystallography has revealed a conserved anion‐binding site likely to interact with phospholipid head groups; the P38G mutation is next to this site and could alter protein flexibility and dynamics upon lipid interaction [12]. On the other side of the P2 structure, a binding surface was identified for a second membrane (Figure 8). Here, Arg88 was identified as a central residue initiating the interaction, and a conformational change of the β5‐β6 loop, leading to opening of the β‐barrel, was observed only in the native membrane composition, not with a simple lipid membrane [30]. The latter conformational change was driven by basic residues on the P2 surface and PS and PI lipids on the membrane system.

A clear distinction was observed between WT P2 and its disease variants. The CMT variants, having single mutations in the β‐sheet regions of the small β‐barrel protein (I43N, I52T), exhibited only minor deviations in binding. This correlates with earlier findings [12] that the β5–β6 strands (see Figure 8) of the barrel region are central in lipid membrane binding.

In contrast, the P38G variant displays significant differences in binding to lipid monolayers. This point mutation, substituting proline with glycine in the hinge region between the α‐helical lid and the β‐sheet barrel structure, is noteworthy (Figure 8). It is well‐established that membrane binding induces structural changes in P2, primarily involving the partial unfolding of the α‐helical lid [12, 13] and opening of the β5–β6 loop. P38G was earlier shown to be more dynamic than WT P2, with altered membrane‐binding properties [13, 30, 45]. The altered membrane affinity of the P38G variants observed in monolayer measurements, coupled with faster membrane binding [12, 13], may be attributed to enhanced protein flexibility, facilitating altered lipid interactions. Therefore, the combined data from Laulumaa et al.^.^ [13] and our monolayer measurements suggest that P38G exhibits faster association kinetics but lower dynamic properties locally. This implies that P38G associates with and dissociates from the membrane more rapidly, but with overall lower affinity; remarkably, P38G is the only P2 variant thus far crystallized without any bound fatty acid—suggesting also altered kinetics of fatty acid binding within the barrel in this hinge mutant [30]. The importance of the helical lid region was observed also on other FABPs (FABP3, 4, 5) [25] in solution with regard to its interaction with fatty acids. A prebinding site for fatty acids on the outside of the protein, close to the helical lid, was identified for these three FABPs. Within the cellular context, P2 variants could potentially enhance fatty acid transport by accelerating membrane association, while simultaneously compromising stable membrane stacking, which is crucial for the “solid” permanent structure of the myelin sheath.

The P38G variation in the region between the helical lid and the β‐sheet thus has a significant influence on the membrane affinity also in the complex, native‐like lipid monolayers studied here. Further, two‐color epifluorescence experiments with additional P2 variants could shed light on the interaction between protein localization and membrane affinity. For this, mutations in the helical lid and/or other sites in various regions of the protein would be of great importance. A line of CMT variants of P2 is known [8, 31], and structure‐based mutations [28] should be included in such a study. Considering that all lipid membrane interaction experiments here and in our earlier work were carried out on the human P2 protein, it is important to highlight that mutations in the *PMP2* gene cause human CMT disease, and that unlike the mouse, human brain myelinating cells, in addition to PNS Schwann cells, express P2 protein. Accordingly, while the mouse deficient in *PMP2* had a mild phenotype, P2 could be an example of a protein relevant to understand the differences between mice and humans [7, 8, 9, 13, 14, 34]. It is therefore important to study P2 and the CMT variants described here, in lipid mixtures mimicking the CNS myelin composition of humans as well, which is planned in our laboratories for the future.

Another approach toward understanding native myelin at the molecular level is to further increase the system complexity. We have here shown affinity toward complex lipid mixtures, which is in line with previous publications [7, 8, 12, 13, 30]. However, since P2 does not occur alone in the myelin sheath, a combination of P2 with the myelin proteins MBP/P1 [57], PMP22, and P0, possibly with the help of lipid nanodiscs [70], would be a considerable further development of the system. Accordingly, we recently looked at the possible synergy and competition between P2, MBP, and the cytoplasmic domain of P0 [30]. All three proteins share the function of stacking lipid bilayers at the PNS myelin major dense line, despite their unrelated structure. In follow‐up studies on complex proteolipid systems, not only the single protein–lipid interaction—including disease variants—will be demonstrated, along with synergistic or antagonistic effects of the myelin proteins on protein–lipid interactions. Further research with increasing protein complexity in myelin model systems is currently in progress and will shed light on the molecular foundation of myelin and its formation.

## Supporting Information

Additional Supporting Information can be found online in the Supporting Information section. Adsorption isotherms, MIP plots, compression and adsorption isotherms from fluorescence microscopy experiments, additional epifluorescence images, single‐cell transcriptomics maps, links to the transcriptomics analysis URLs. The authors have cited additional references within the Supporting Information [30].

## Funding

This work was supported by Deutsche Forschungsgemeinschaft (grant 436494874), Norges Forskningsråd (grants 324877, 245828, 245922).

## Conflicts of Interest

The authors declare no conflicts of interest.

## Supporting information

Supplementary Material

## Data Availability

The data that support the findings of this study are available from the corresponding author upon reasonable request.
