# Supplementary material for: Human Peripheral Myelin Protein 2 and Charcot–Marie–Tooth Disease or Structural Missense Variants Show Different Binding to Myelin‐Like Lipid Monolayers
Source: Chembiochem. 2026 Apr 21;27(8):e202500947. doi: 10.1002/cbic.202500947 (PMC13096861; doi:10.1002/cbic.202500947)
Supplement: Supplementary file 1 — Supplementary Material [file CBIC-27-e202500947-s001.pdf]

Human Peripheral Myelin Protein 2 and Charcot-Marie-Tooth Disease or Structural Missense Variants Show Different Binding to Myelin-Like Lipid Monolayers

- Supporting Information -

Florian Arndt Schöffmann[1], Md Abdus Shukur Imran[2], Christian Schwieger[1], Øystein Hetland[3], Vanessa Jerschabek[1], Arne Raasakka[3], Petri Kursula[2,3,4], Dariush Hinderberger[1]

[1] *Institute of Chemistry, Physical Chemistry – Complex Self-Organizing Systems, Martin-Luther-Universität Halle-Wittenberg, Von-Danckelmann-Platz 4, 06120 Halle (Saale), Germany*

[2] *Biocenter Oulu and Faculty of Biochemistry and Molecular Medicine, University of Oulu, Aapistie 7, 90220 Oulu, Finland*

[3] *Department of Biomedicine, University of Bergen, Jonas Lies vei 91, 5009 Bergen, Norway*

[4] *LINXS Institute of Advanced Neutron and X-Ray Science, Scheelevägen 19, 22370 Lund, Sweden*

Corresponding author: Dariush Hinderberger, Email: dariush.hinderberger@chemie.uni-halle.de

Fluid Mosaic Model

Since Singer & Nicolson[62] published the F-MMM in 1972, it has lost some of its significance for membrane science. In 2014, Nicolson[63] updated this model to include the latest findings in membrane/lipid research. Basically, the driving factors for the assembly of the lipid bilayer are the hydrophobic effect and van der Waals forces, which lead to the displacment of water from the lipid structures. Nicolson, with reference to Mouritsen's[64,65] publications, describes the role of cholesterol as 'schizophrenic'. Since it is equally present in the liquid and solid phases, Mouritsen[64,65] postulated the new type of membrane phase as a liquid-ordered (Lo) phase. This is accompanied by the lipid raft hypothesis[66], which describes these specialized domains and their differentiation. The process of raft formation is described as highly dynamic and reversible, driven primarily by hydrophobic and van der Waals forces, as well as by hydrogen bonds and charge pairing, especially in the case of charged phospholipids and sphingolipids.

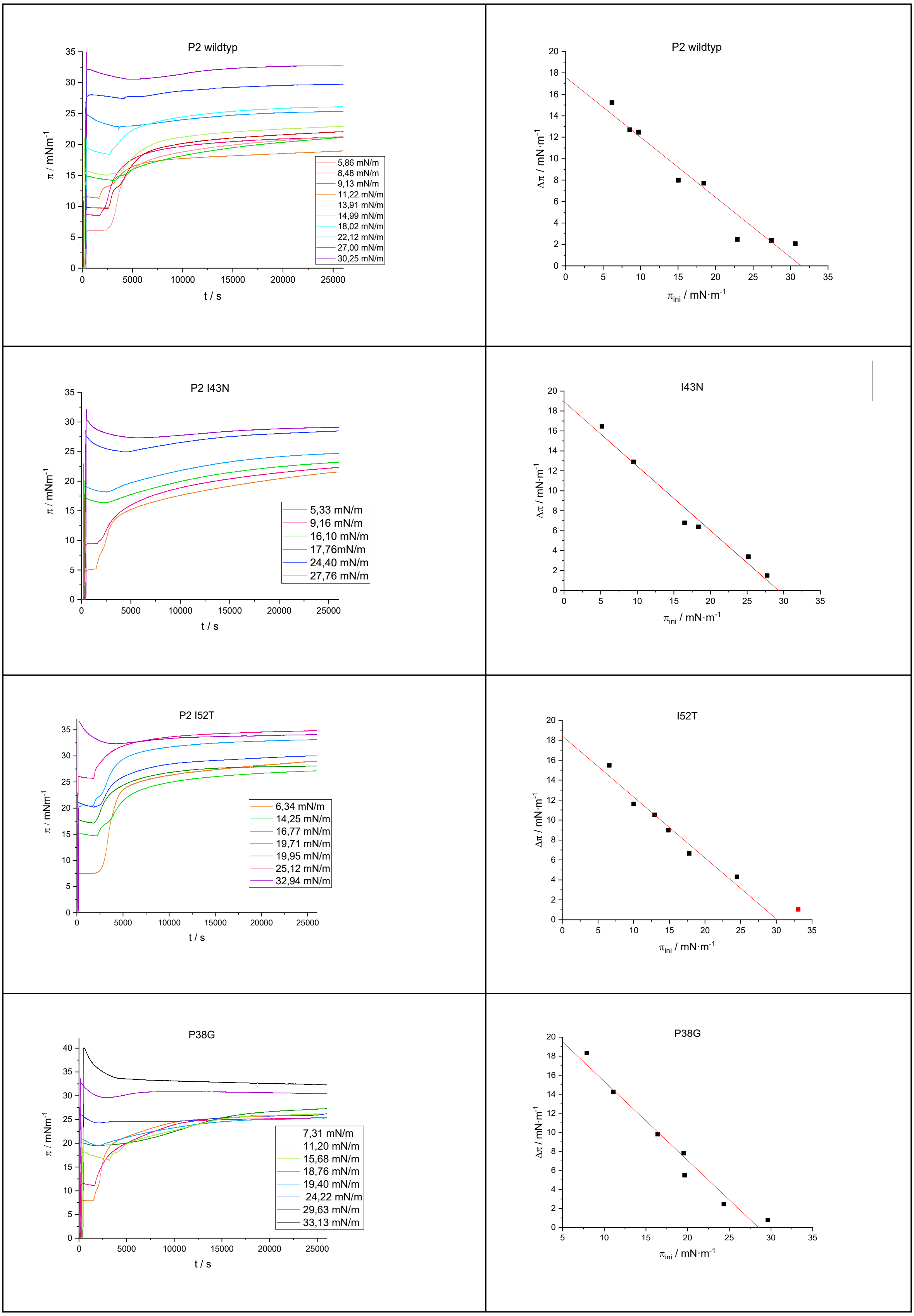

Figure S1. Left: Surface pressure measurements on a Langmuir film balance with a constant surface upon injection of P2 variants underneath a PNS-myelin-like lipid monolayer with variable initial surface pressures. Right: Plot of surface pressure increase (Dp) upon adsorption of the respective proteins vs. initial surface pressure (p_{ini}) of the lipid monolayer and linear regression (red line); top to button: P2 wildtype; P2 I43N; P2 I52T; P2 P38G.

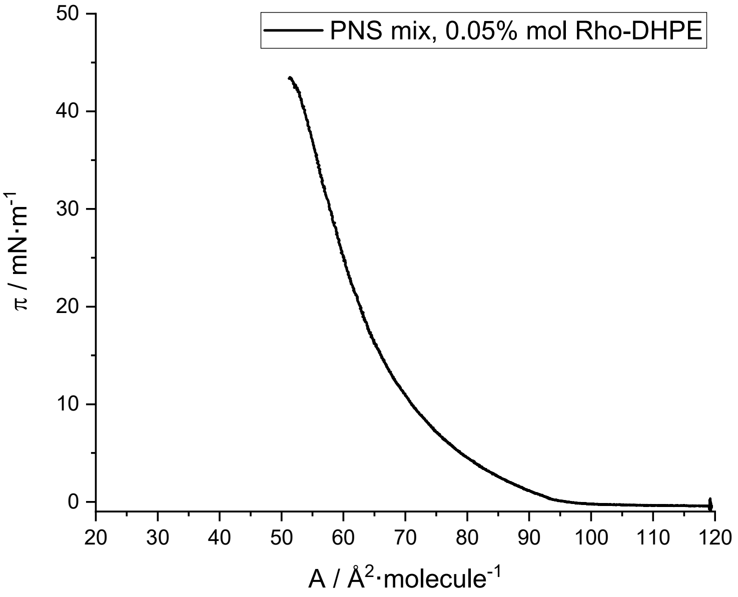

Figure S2. Surface pressure - area isotherm of a PNS-myelin-like monolayer including 0.05 mol% Rho-DHPE on a HEPES-NaCl buffer subphase at 20 °C.

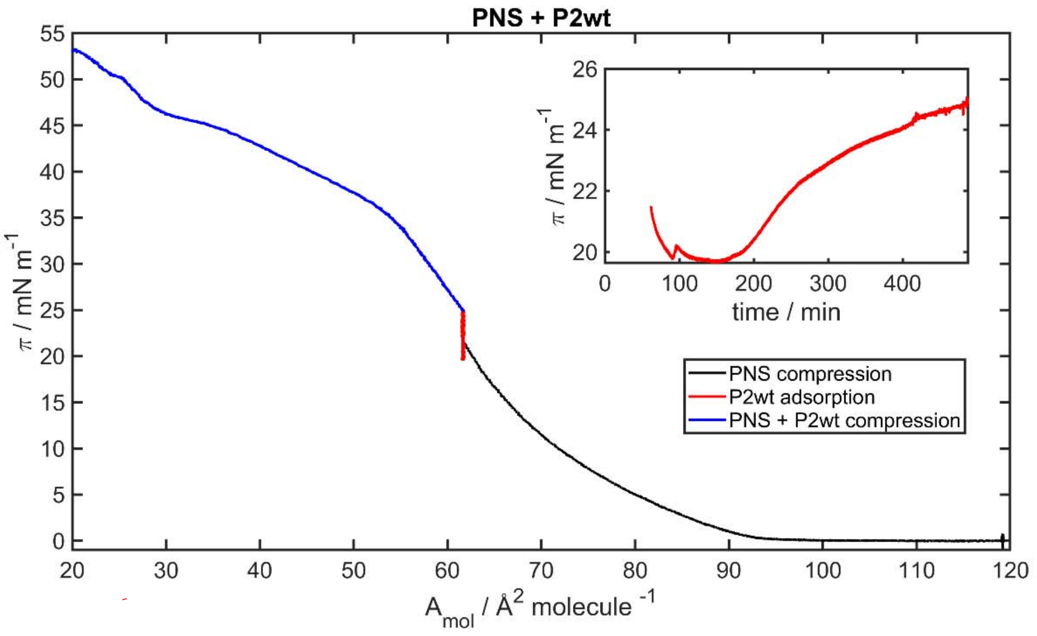

Figure S3. Isotherm showing the compression behavior of a PNS-myelin-like monolayer mixed with 0.05% Rho-DHPE on HEPES-NaCl buffer subphase (black) as well as the compression after adsorption of P2 wt and P2 wt -Alexa-488-conjugate (blue). The proteins were injected at a surface pressure of 20 mN/m and allowed to adsorb at constant area (red). The inset shows the time course of adsorption. Epifluorescence images recorded during compression and adsorption are shown in **Figure 3.**

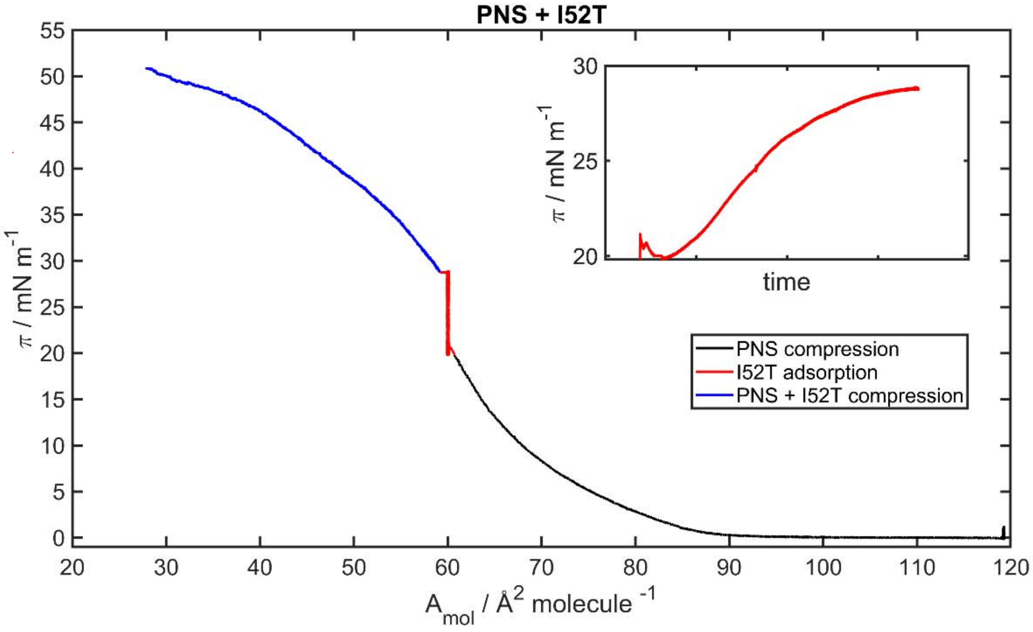

Figure S4. Isotherm showing the compression behavior of a PNS-myelin-like monolayer mixed with 0.05% Rho-DHPE on HEPES-NaCl buffer subphase (black) as well as the compression after adsorption of P2 I52T (blue). The protein was injected at a surface pressure of 20 mN/m and allowed to adsorb at constant area (red). The inset shows the time course of adsorption.

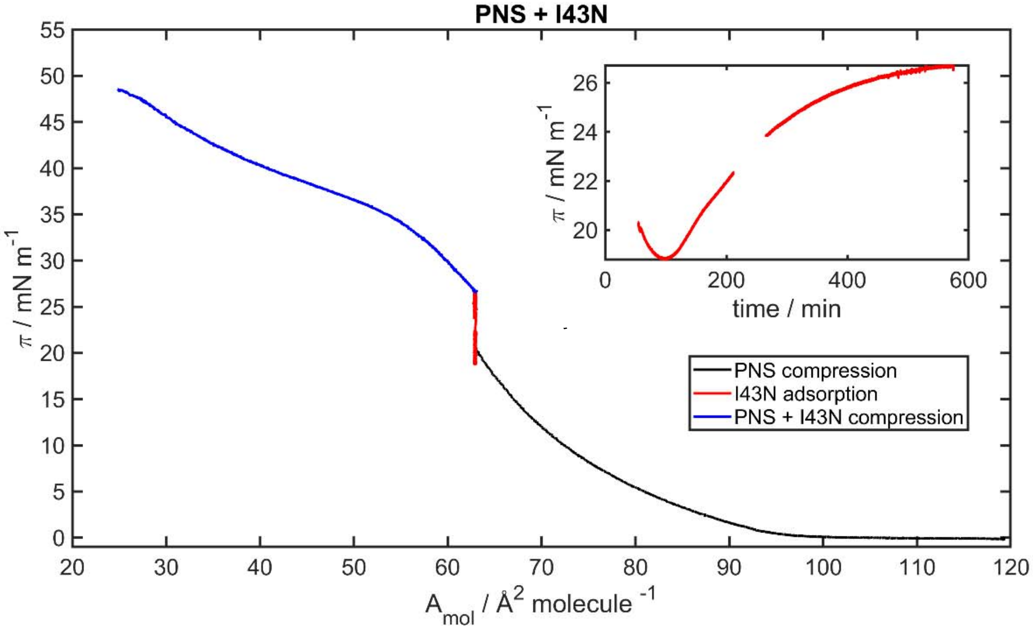

Figure S5. Isotherm showing the compression behavior of a PNS-myelin-like monolayer mixed with 0.05% Rho-DHPE on HEPES-NaCl buffer subphase (black) as well as the compression after adsorption of P2 I43N (blue). The protein was injected at a surface pressure of 20 mN/m and allowed to adsorb at constant area (red). The inset shows the time course of adsorption.

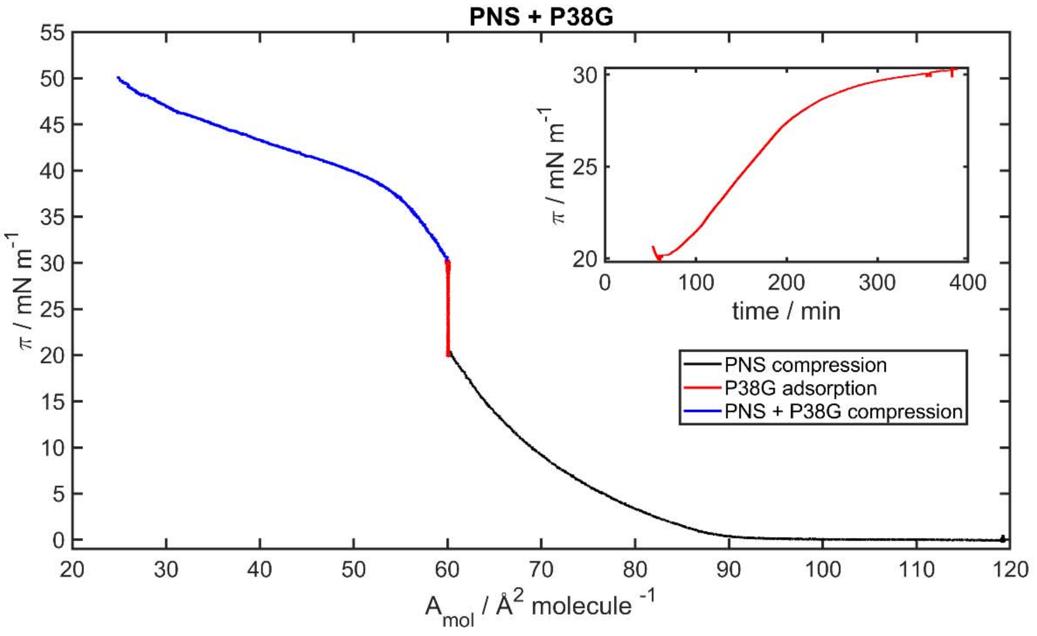

Figure S6. Isotherm showing the compression behavior of a PNS-myelin-like monolayer mixed with 0.05% Rho-DHPE on HEPES-NaCl buffer subphase (black) as well as the compression after adsorption of P2 P38G (blue). The protein was injected at a surface pressure of 20 mN/m and allowed to adsorb at constant area (red). The inset shows the time course of adsorption. Fluorescence images recorded during compression and adsorption are shown in **Figure 4**.

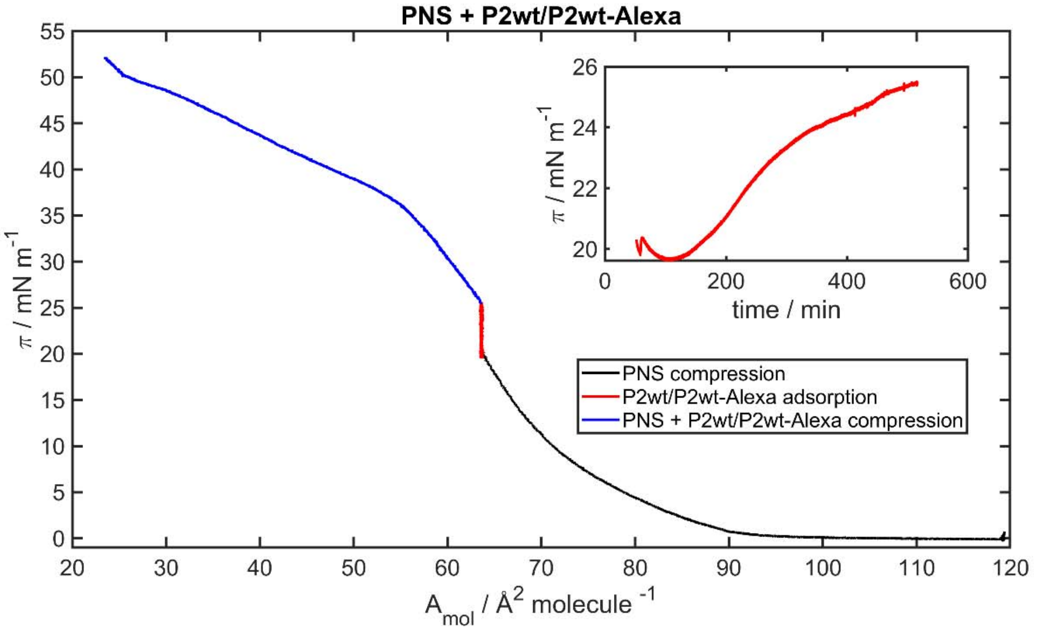

Figure S7. Isotherm showing the compression behavior of a PNS-myelin-like monolayer mixed with 0.05% Rho-DHPE on HEPES-NaCl buffer subphase (black) as well as the compression after adsorption of P2wt and P2wt-Alexa-488-conjugate (blue). The proteins were injected at a surface pressure of 20 mN/m and allowed to adsorb at constant area (red). The inset shows the time course of adsorption. Epifluorescence images recorded during compression and adsorption are shown in **Figure 3 and Figure 5 A-C.**

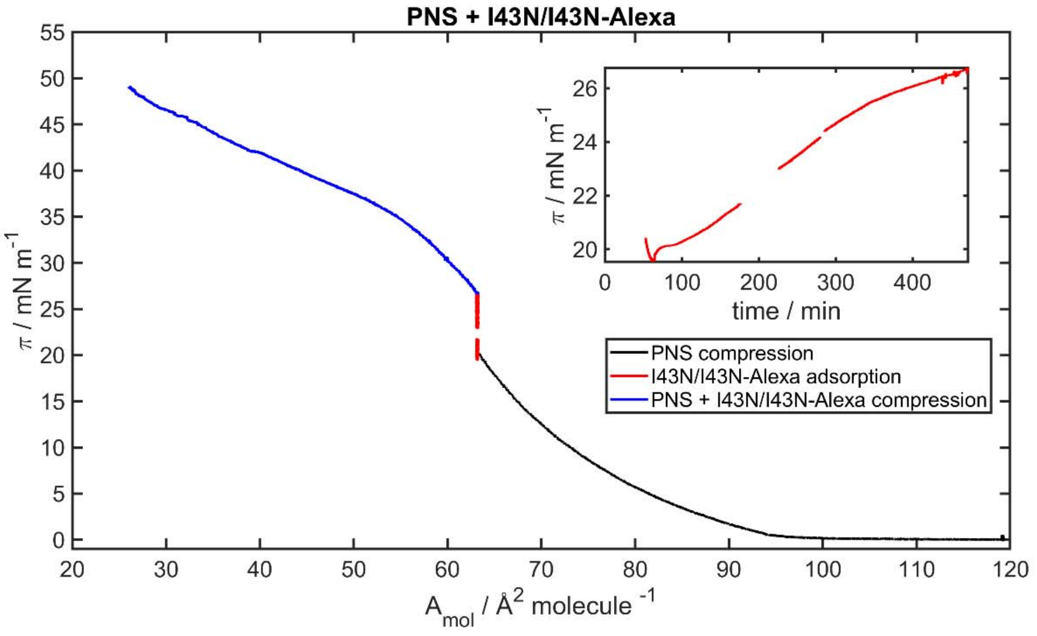

Figure S8. Isotherm showing the compression behavior of a PNS-myelin-like monolayer mixed with 0.05% Rho-DHPE on HEPES-NaCl buffer subphase (black) as well as the compression after adsorption of P2 I43N and P2 I43N -Alexa-488-conjugate (blue). The proteins were injected at a surface pressure of 20 mN/m and allowed to adsorb at constant area (red). The inset shows the time course of adsorption. Epifluorescence images recorded during compression and adsorption are shown in **Figure S12.**

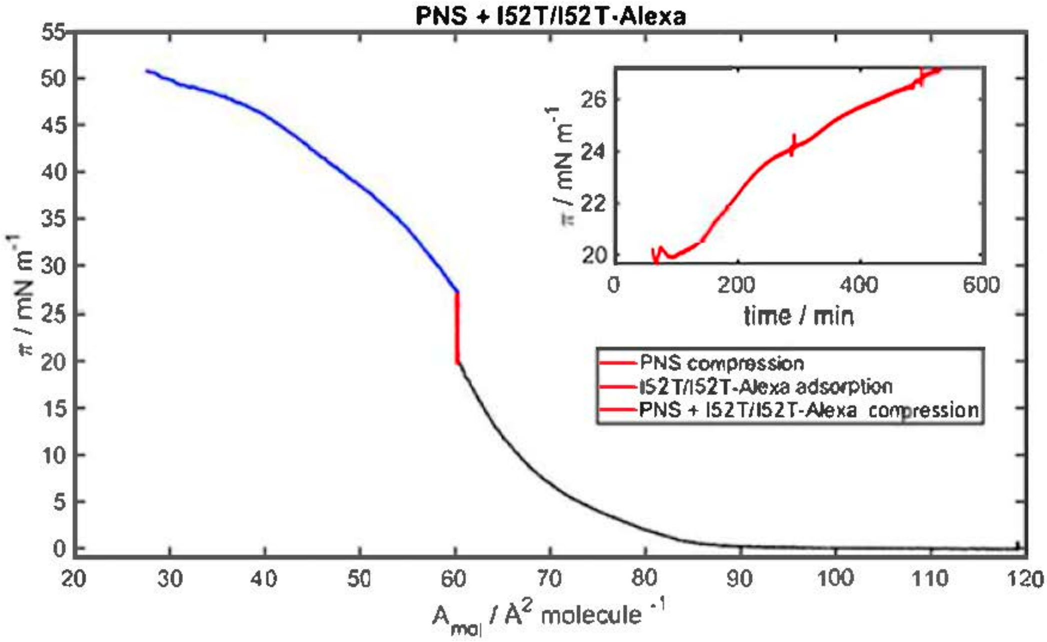

Figure S9. Isotherm showing the compression behavior of a PNS-myelin-like monolayer mixed with 0.05% Rho-DHPE on HEPES-NaCl buffer subphase (black) as well as the compression after adsorption of P2 I52T and P2 I52T -Alexa-488-conjugate (blue). The proteins were injected at a surface pressure of 20 mN/m and allowed to adsorb at constant area (red). The inset shows the time course of adsorption. Epifluorescence images recorded during compression and adsorption are shown in **Figure S13.**

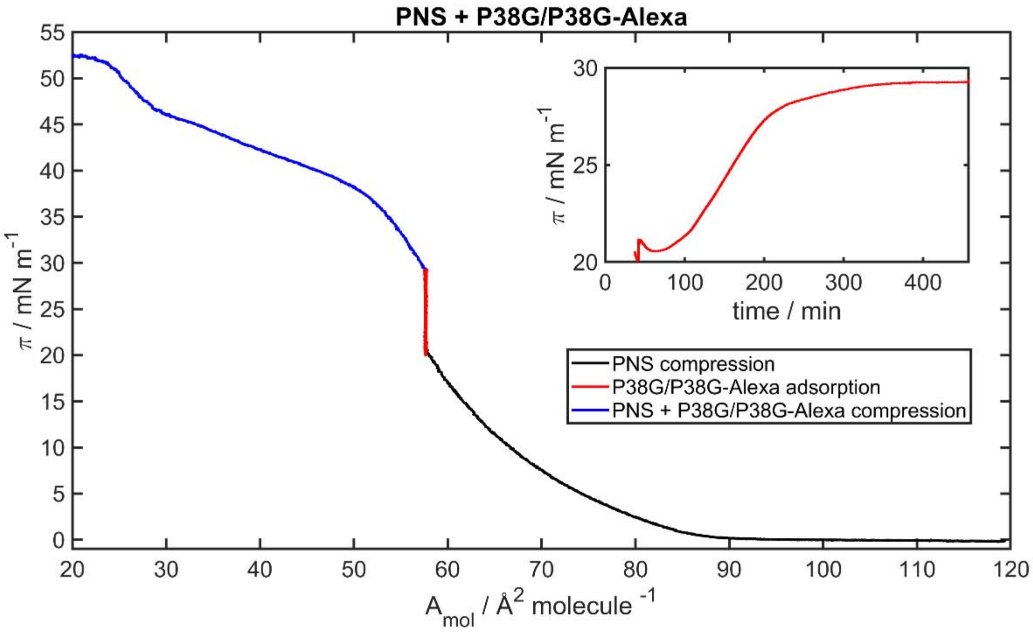

Figure S10. Isotherm showing the compression behavior of a PNS-myelin-like monolayer mixed with 0.05% Rho-DHPE on HEPES-NaCl buffer subphase (black) as well as the compression after adsorption of P2 P38G and P2 IP38G-Alexa-488-conjugate (blue). The proteins were injected at a surface pressure of 20 mN/m and allowed to adsorb at constant area (red). The inset shows the time course of adsorption. Epifluorescence images recorded during compression and adsorption are shown in **Figure 4 and Figure 5 D-F**.

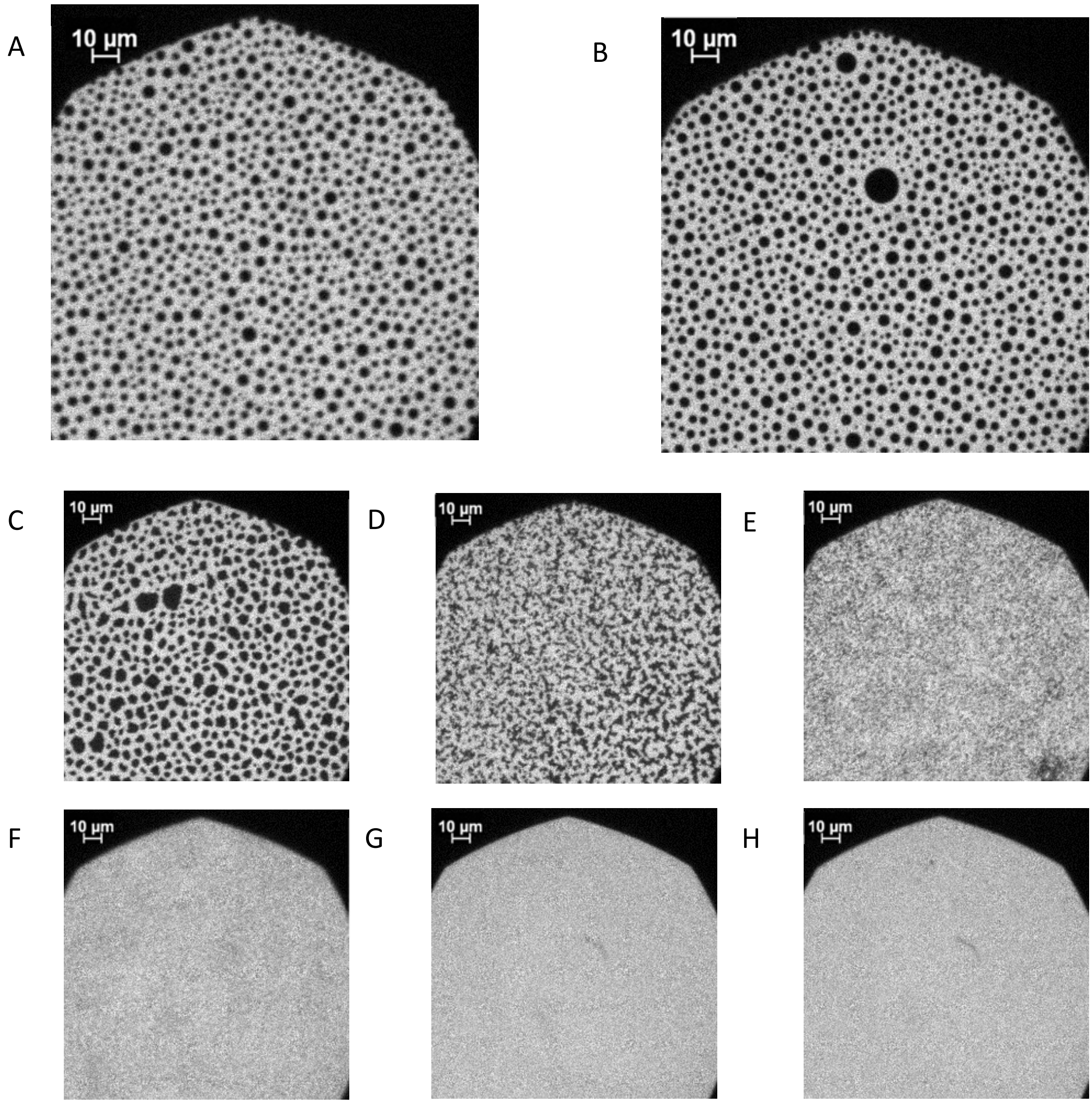

Figure S11. Representative epifluorescence microscopy images of the PNS-myelin-like monolayer mixed with 0.05 mol% Rho-DHPE on a HEPES-NaCl buffer subphase; A: 0 mN/m; B: 1.1 mN/m; C: 11.3 mN/m; D: 11.6 mN/m; E: 12.8 mN/m; F: 25.1 mN/m; G: 33 mN/m; H: 40.52 mN/m. The corresponding compression isotherm is shown in **Figure S2**.

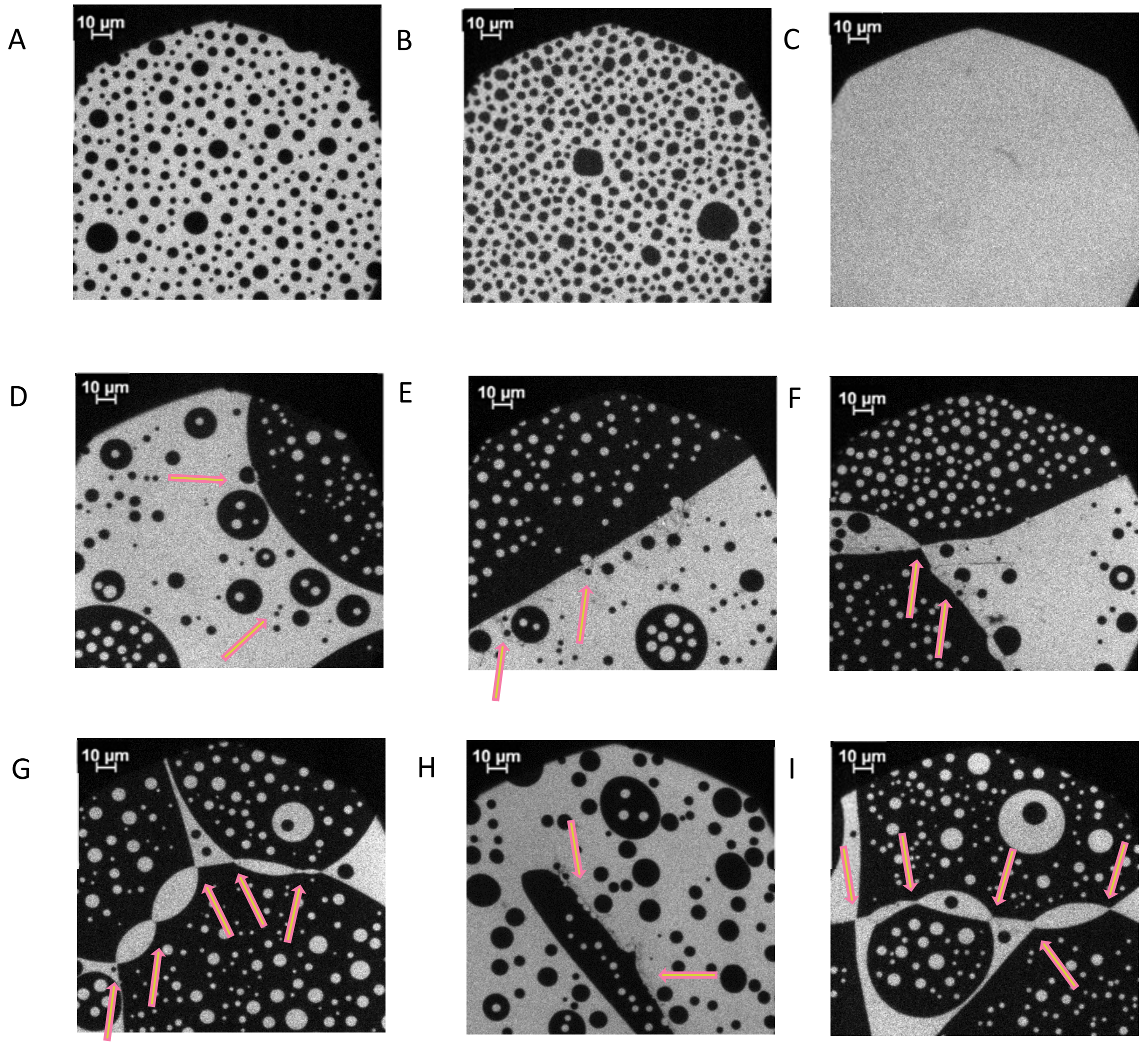

Figure S12. Representative epifluorescence microscopy images of PNS-myelin-like monolayer mixed with 0.05 mol% Rho-DHPE on a HEPES-NaCl buffer subphase before (A-C) and after injection of P2 I43N variant at 20 mN/m and incubation (D-I); A: 2.78 mN/m; B: 5.45 mN/m; C: 12.43 mN/m; D: 17.42 mN/m; E - I: ~ 25 mN/m; Arrows indicate regions of reduced Rho-DHPE fluorescence ("grey veil") due to clusters including the respective P2 variants. The respective Isotherm is shown in **Figure S5**.

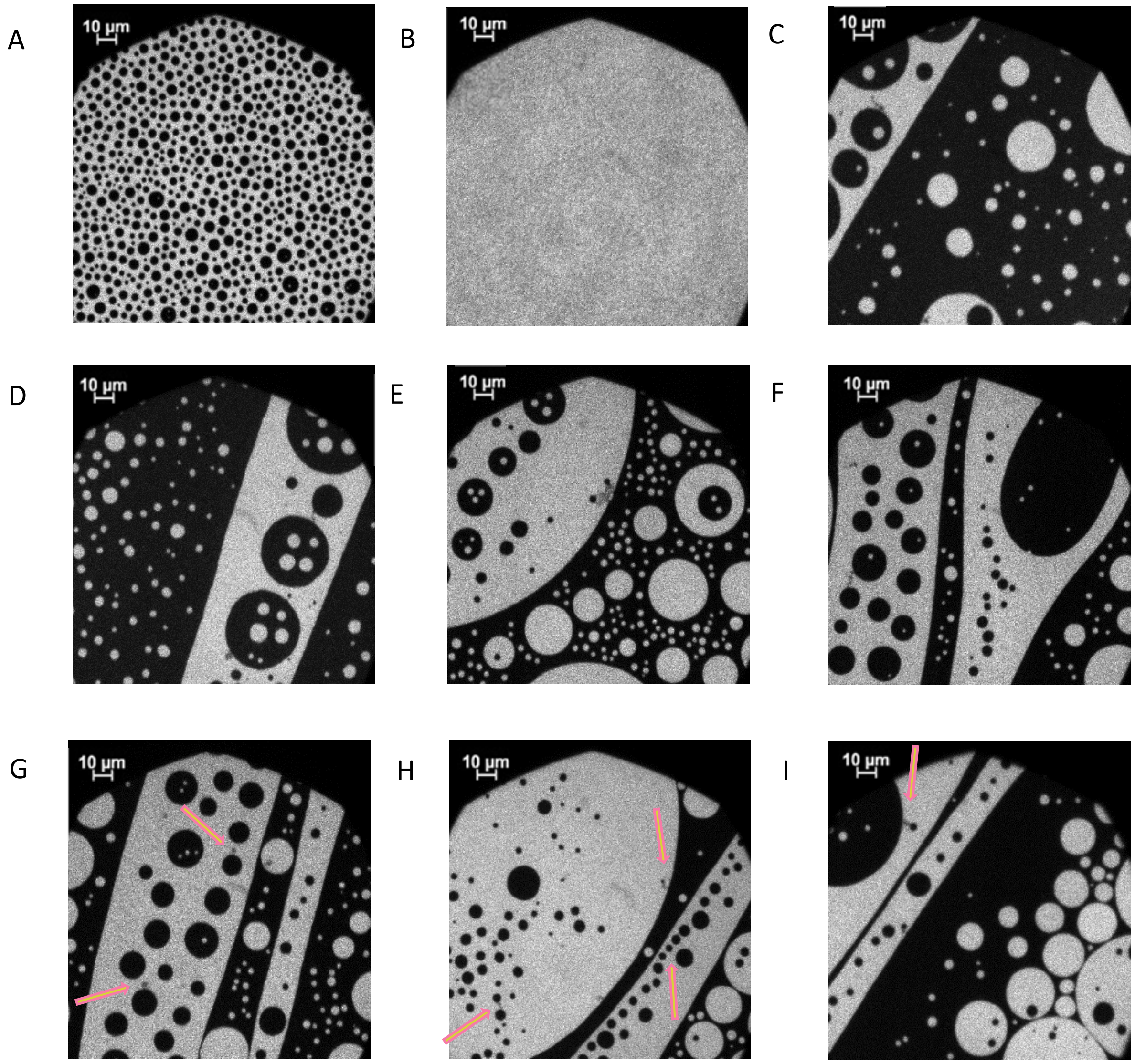

Figure S13. Representative epifluorescence microscopy images of the compression behavior of the PNS-myelin-like monolayer mixed with 0.05 mol% Rho-DHPE on a HEPES-NaCl buffer subphase before (A, B) and after (C – I) injection of P2 I52T variant at 20 mN/m and incubation; A: 5.52 mN/m; B: 9.02 mN/m; C- I: ~ 25 mN/m. Arrows indicate regions of reduced Rho-DHPE fluorescence ("grey veil") due to clusters including the respective P2 variants. The corresponding isotherm is shown in **Figure S4**.

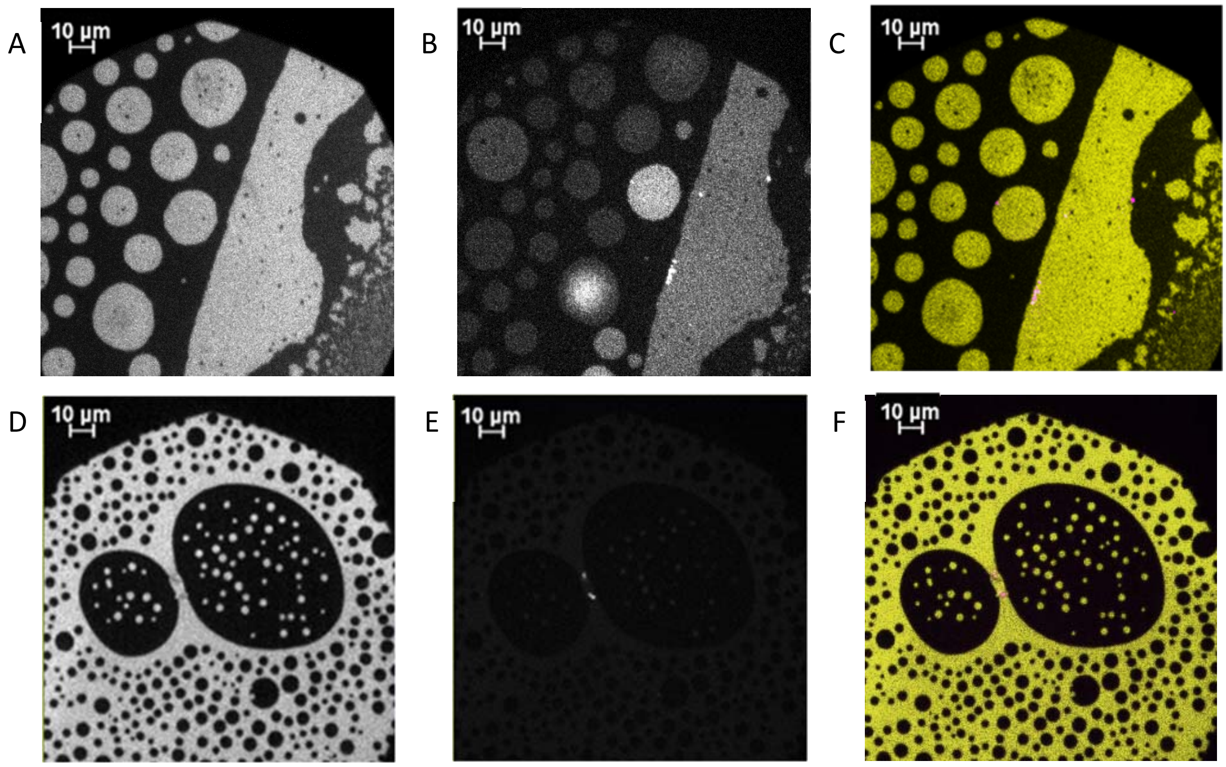

Figure S14. Two-color experiment of P2 variants in epifluorescence film balance microscopy. A, B, C: P2 I52T; D, E, F: P2 I43N; A, D green channel (546 nm excitation wavelength); B, E: blue channel (486 nm excitation wavelength) in myelin-like monolayers at 25 mN/m; C, F: merged images, false coloring for better differentiation: green channel in yellow and blue channel in magenta. The corresponding compression and adsorption isotherms are shown in **Figure S9** (P2 I52T) and **Figure S8** (P2 I43N).

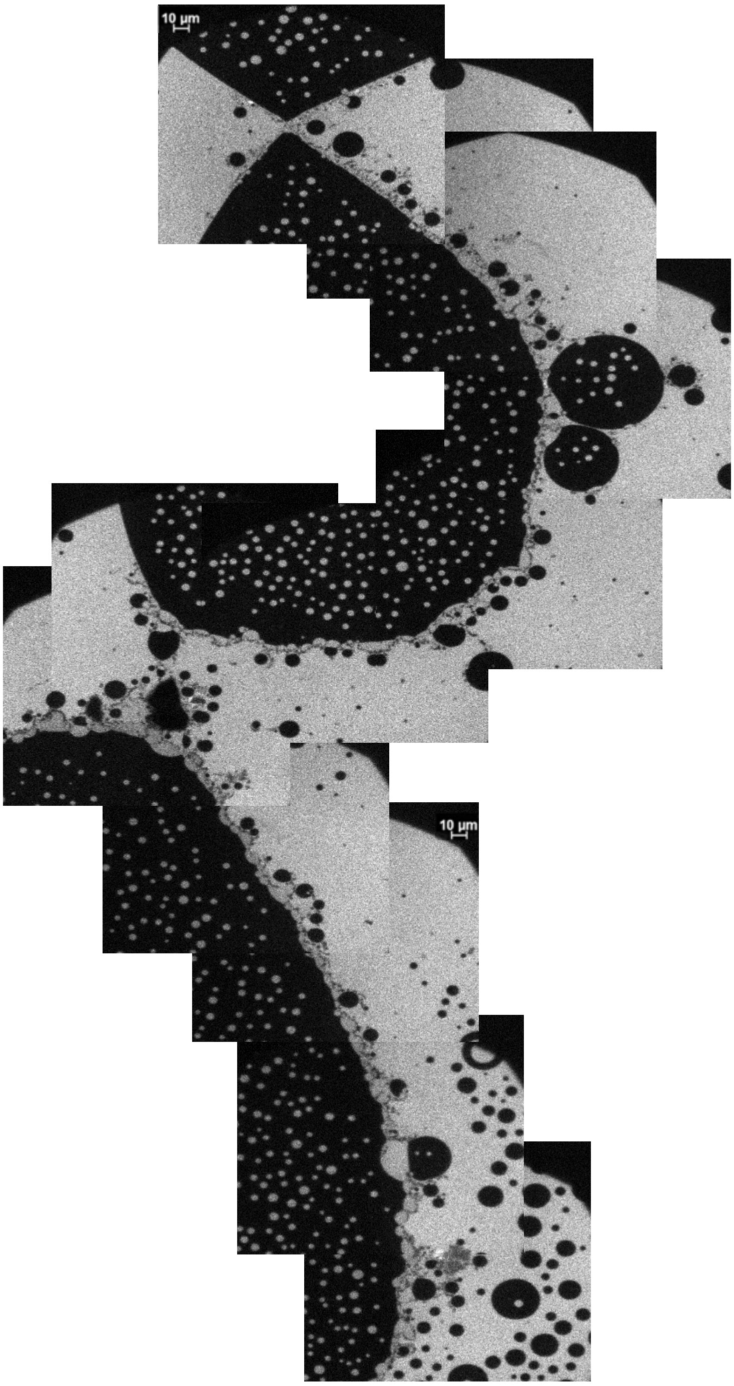

Figure S15. Epifluorescence microscopy of wild-type P2 adsorbed to a PNS-myelin like monolayer marked with Rh-DHPE (isotherms see **Figure S3**). Image series to illustrate the complex cross-linking of the respective domains by the protein.

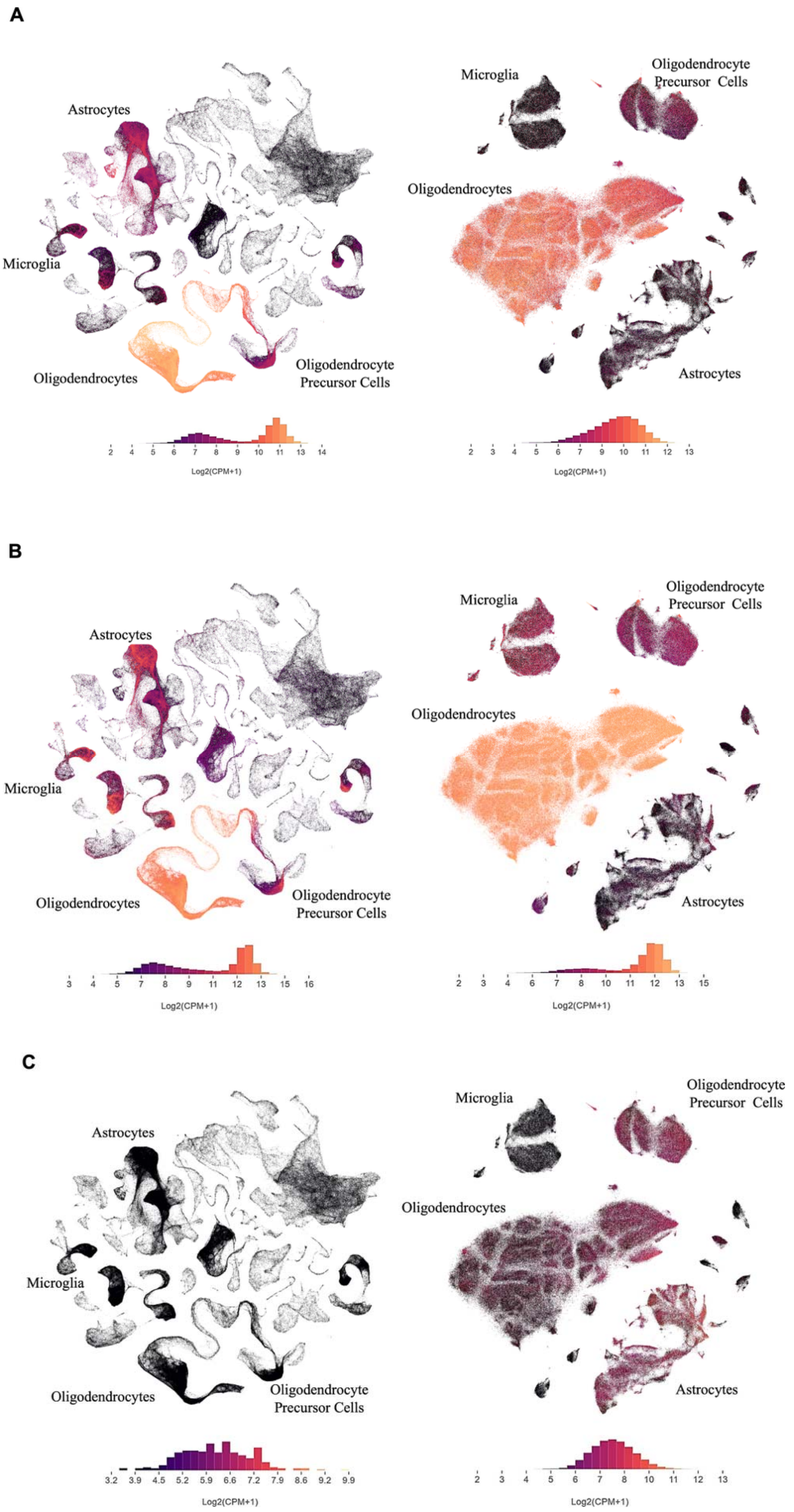

Figure S16. Single-cell transcriptomics maps to complement Figure 7. (A-C) Gene expression in mouse (left) and human (right) non-neuronal cells: (A) *CNP*, (B) *MBP*, and (C) *PMP2*. Mouse 10x scRNA-seq NN-IMN-GC vs human NN datasets were analyzed from ABC Brain Knowledge Platform.

Data Visualization Links:

PMP2 in human non neural cells vs mouse 10x scRNAseq NN-IMN-GC

https://knowledge.brain-map.org/abcatlas#AQIBQVA4Sk5ONUxZQUJHVk1HS1kxQgACTFRNVlBHSlVQWjNLVUhFVUFFUAADAAQBAQKBQJJBgBLlggOEAhllhLJGsAAFAQFQbXAyAAAGAQACUG1wMgADfgAAAAQABYCIGJqCdiQ9BurKAwcCI0ZGRkZGRgADAQZHRU5FAAcACAIAAAg3UFM1RTU3VlA1VFE2SEUzVkVEAAlMVkRCSkFXOEJJNVlTUzFRVUJHAAoACwFub25lAAJub25lAAMBBAEAAiMwMDAwMDAAA8gBAAUBAQIjMDAwMDAwAAPIAQAAAAE5TTY0SjdTMEEzWk5JSkE0VlM2AAJSNUZXVVZURUEyT1c5TkpRTVk5AAMABAEBAneUSn6B34FdA4XGfbWGIMwIAAUBAVBNUDIAAAYBAAJQTVAyAAN%2BAAAABAAFf%2BizrIKafZsGuNoEBwIjRkZGRkZGAAMBBkdFTkUABwAIAgAACDVUNEMzTUMzTE5aV0VCREZVRVoACUMzUlJWQUsxOEhHNlExSk42WlEACgALAW5vbmUAAm5vbmUAAwEEAQACIzAwMDAwMAADyAEABQEBAiMwMDAwMDAAA8gBAAAAAgMA

PMP2 OPC Oligo

https://knowledge.brain-map.org/abcatlas#AQIBQVA4Sk5ONUxZQUJHVk1HS1kxQgACTFRNVlBHSlVQWjNLVUhFVUFFUAADBAFGUzAwRFhWMFQ5UjFYOUZKNFFFAAlBMzEgT1BDLU9saWdvAAABUVk1UzhLTU81SExKVUYwUDAwSwACAAABMTVCSzQ3RENJT0YxU0xMVVc5UAACAAABQ0JHQzBVMzBWVjlKUFI2MFRKVQACAAAEAQECgcl6bYE5rJYDg3CidYPyYV4ABQEBUG1wMgAABgEAAlBtcDIAA34AAAAEAAWAiBiagnYkPQbqygMHAiNGRkZGRkYAAwAGR0VORQAHAAgCAAAIN1BTNUU1N1ZQNVRRNkhFM1ZFRAAJTFZEQkpBVzhCSTVZU1MxUVVCRwAKAAsBbm9uZQACbm9uZQADAQQBAAIjMDAwMDAwAAPIAQAFAQECIzAwMDAwMAADyAEAAAABOU02NEo3UzBBM1pOSUpBNFZTNgACUjVGV1VWVEVBMk9XOU5KUU1ZOQADAwFYM0s3SzJFRzZVTVhPTjBUOUJFAAICT2xpZ29kZW5kcm9jeXRlAE9saWdvZGVuZHJvY3l0ZSBwcmVjdXJzb3IAAAFNWEVGT0ZOU1RNU0ZSSEZORDE5AAIAAAFCUjlOU1NXNEhGWVhUQlVISFIzAAIAAAQBAQKBAQ5agvK6SgOFCrp%2FhcLYZwAFAQFQTVAyAAAGAQACUE1QMgADfgAAAAQABX%2Fos6yCmn2bBrjaBAcCI0ZGRkZGRgADAAZHRU5FAAcACAIAAAg1VDRDM01DM0xOWldFQkRGVUVaAAlDM1JSVkFLMThIRzZRMUpONlpRAAoACwFub25lAAJub25lAAMBBAEAAiMwMDAwMDAAA8gBAAUBAQIjMDAwMDAwAAPIAQAAAAIDAA%3D%3D

CNP in human non neural cells vs mouse 10x scRNAseq NN-IMN-GC

https://knowledge.brain-map.org/abcatlas#AQIBQVA4Sk5ONUxZQUJHVk1HS1kxQgACTFRNVlBHSlVQWjNLVUhFVUFFUAADAAQBAQKBqkm7gXaMlwOEHrqehPjqhAAFAQFDbnAAAAYBAAJDbnAAA34AAAAEAAWAVuI%2FgpBstwbq4gMHAiNGRkZGRkYAAwEGR0VORQAHAAgCAAAIN1BTNUU1N1ZQNVRRNkhFM1ZFRAAJTFZEQkpBVzhCSTVZU1MxUVVCRwAKAAsBbm9uZQACbm9uZQADAQQBAAIjMDAwMDAwAAPIAQAFAQECIzAwMDAwMAADyAEAAAABOU02NEo3UzBBM1pOSUpBNFZTNgACUjVGV1VWVEVBMk9XOU5KUU1ZOQADAAQBAQKBEHt8f1HnAgOG6K8BhTZN4wAFAQFDTlAAAAYBAAJDTlAAA34AAAAEAAV%2FLOnWgtaRowbSwgYHAiNGRkZGRkYAAwEGR0VORQAHAAgCAAAINVQ0QzNNQzNMTlpXRUJERlVFWgAJQzNSUlZBSzE4SEc2UTFKTjZaUQAKAAsBbm9uZQACbm9uZQADAQQBAAIjMDAwMDAwAAPIAQAFAQECIzAwMDAwMAADyAEAAAACAwA%3D

MBP in human non neural cells vs mouse 10x scRNAseq NN-IMN-GC

https://knowledge.brain-map.org/abcatlas#AQIBQVA4Sk5ONUxZQUJHVk1HS1kxQgACTFRNVlBHSlVQWjNLVUhFVUFFUAADAAQBAQKBqkm7gXaMlwOEHrqehPjqhAAFAQFNYnAAAAYBAAJNYnAAA34AAAAEAAWAOolPgh7A%2Bwa0DgcCI0ZGRkZGRgADAQZHRU5FAAcACAIAAAg3UFM1RTU3VlA1VFE2SEUzVkVEAAlMVkRCSkFXOEJJNVlTUzFRVUJHAAoACwFub25lAAJub25lAAMBBAEAAiMwMDAwMDAAA8gBAAUBAQIjMDAwMDAwAAPIAQAAAAE5TTY0SjdTMEEzWk5JSkE0VlM2AAJSNUZXVVZURUEyT1c5TkpRTVk5AAMABAEBAoEQe3x%2FUecCA4borwGFNk3jAAUBAU1CUAAABgEAAk1CUAADfgAAAAQABX9l%2F%2FOC0J%2FRBrjJBAcCI0ZGRkZGRgADAQZHRU5FAAcACAIAAAg1VDRDM01DM0xOWldFQkRGVUVaAAlDM1JSVkFLMThIRzZRMUpONlpRAAoACwFub25lAAJub25lAAMBBAEAAiMwMDAwMDAAA8gBAAUBAQIjMDAwMDAwAAPIAQAAAAIDAA%3D%3D

PMP2 in human nn vs neuron

https://knowledge.brain-map.org/abcatlas#AQIBOU02NEo3UzBBM1pOSUpBNFZTNgACR1o5NFo4M1JYVVRFQ0hZS1FPNAADAAQBAQKAZHJPgVS7DwOFrOnqhchczAAFAQFQTVAyAAAGAQACUE1QMgADfgAAAAQABX%2Fos6yCmn2bBrjaBAcCI0ZGRkZGRgADAAZHRU5FAAcACAIAAAhVTVpKN1pMRjhHNUZlNVM5WTRVAAlDM1JSVkFLMThIRzZRMUpONlpRAAoACwFub25lAAJub25lAAMBBAEAAiMwMDAwMDAAA8gBAAUBAQIjMDAwMDAwAAPIAQAAAAE5TTY0SjdTMEEzWk5JSkE0VlM2AAJSNUZXVVZURUEyT1c5TkpRTVk5AAMABAEBAn83udeA2Ro8A4VKHvmFFK%2FZAAUBAVBNUDIAAAYBAAJQTVAyAAN%2BAAAABAAFf%2BizrIKafZsGuNoEBwIjRkZGRkZGAAMABkdFTkUABwAIAgAACDVUNEMzTUMzTE5aV0VCREZVRVoACUMzUlJWQUsxOEhHNlExSk42WlEACgALAW5vbmUAAm5vbmUAAwEEAQACIzAwMDAwMAADyAEABQEBAiMwMDAwMDAAA8gBAAAAAgMA

MPZ in human non neural cells vs mouse 10x scRNAseq NN-IMN-GC

https://knowledge.brain-map.org/abcatlas#AQIBOU02NEo3UzBBM1pOSUpBNFZTNgACUjVGV1VWVEVBMk9XOU5KUU1ZOQADAAQBAQKC9Bh5eVJIXQOGkiCNhs5pdAAFAQFNUFoAAAYBAAJNUFoAA34AAAAEAAV%2F%2FFHhglhOzQbgoAQHAiNGRkZGRkYAAwEGR0VORQAHAAgCAAAINVQ0QzNNQzNMTlpXRUJERlVFWgAJQzNSUlZBSzE4SEc2UTFKTjZaUQAKAAsBbm9uZQACbm9uZQADAQQBAAIjMDAwMDAwAAPIAQAFAQECIzAwMDAwMAADyAEAAAABQVA4Sk5ONUxZQUJHVk1HS1kxQgACTFRNVlBHSlVQWjNLVUhFVUFFUAADAAQBAQKAKoVogaJZVgOEIFx%2BhKCQZgAFAQFNcHoAAAYBAAJNcHoAA34AAAAEAAWATDhygpYraAbejwEHAiNGRkZGRkYAAwEGR0VORQAHAAgCAAAIN1BTNUU1N1ZQNVRRNkhFM1ZFRAAJTFZEQkpBVzhCSTVZU1MxUVVCRwAKAAsBbm9uZQACbm9uZQADAQQBAAIjMDAwMDAwAAPIAQAFAQECIzAwMDAwMAADyAEAAAACAwA%3D

References

[71] G. L. Nicolson, "The Fluid - Mosaic Model of Membrane Structure: Still relevant to understanding the structure, function and dynamics of biological membranes after more than 40 years.", *Biochim. Biophys. Acta - Biomembr.* **2014**, *1838*, 1451–1466.

[72] O. G. Mouritsen, "Model answers to lipid membrane questions.", *Cold Spring Harb. Perspect. Biol.* **2011**, *3*, a004622.

[73] K. Jacobson, O. G. Mouritsen, R. G. W. Anderson, "Lipid rafts: At a crossroad between cell biology and physics.", *Nat. Cell Biol.* **2007**, *9*, 7–14.

[74] K. Simons, E. Ikonen, "Functional rafts in cell membranes.", *Nature* **1997**, *387*, 569–572.
